# Programmed neurite degeneration in human central nervous system neurons driven by changes in NAD^+^ metabolism

**DOI:** 10.1038/s41419-024-07326-w

**Published:** 2025-01-17

**Authors:** Markus Brüll, Selina Multrus, Michael Schäfer, Ivana Celardo, Christiaan Karreman, Marcel Leist

**Affiliations:** 1 In vitro Toxicology and Biomedicine, Dept. inaugurated by the Doerenkamp-Zbinden foundation, University of Konstanz, 78457 Konstanz, Germany; 2 CAAT-Europe, University of Konstanz, 78457 Konstanz, Germany

**Keywords:** Cell death in the nervous system, Neurodegeneration

## Abstract

Neurite degeneration (ND) precedes cell death in many neurodegenerative diseases. However, it remains unclear how this compartmentalized cell death process is orchestrated in the central nervous system (CNS). The establishment of a CNS axotomy model (using modified 3D LUHMES cultures) allowed us to study metabolic control of ND in human midbrain-derived neurons without the use of toxicants or other direct disturbance of cellular metabolism. Axotomy lead to a loss of the NAD^+^ synthesis enzyme NMNAT2 within 2 h and a depletion of NAD^+^ within 4-6 h. This process appeared specific, as isolated neurites maintained ATP levels and a coupled mitochondrial respiration for at least 6 h. In the peripheral nervous system (PNS) many studies observed that NAD^+^ metabolism, in particular by the NADase SARM1, plays a major role in the ND occurring after axotomy. Since neither ferroptosis nor necroptosis, nor caspase-dependent apoptosis seemed to be involved in neurite loss, we investigated SARM1 as potential executioner (or controller). Knock-down or expression of a dominant-negative isoform of SARM1 indeed drastically delayed ND. Various modifications of NAD^+^ metabolism known to modulate SARM1 activity showed the corresponding effects on ND. Moreover, supplementation with NAD^+^ attenuated ND. As a third approach to investigate the role of altered NAD^+^ metabolism, we made use of the WLD(s) protein, which has been found in a mutant mouse to inhibit Wallerian degeneration of axons. This protein, which has a stable NMNAT activity, and thus can buffer the loss of NMNAT2, protected the neurites by stabilizing neurite NAD^+^ levels. Thus CNS-type ND was tightly linked to neurite metabolism in multiple experimental setups. Based on this knowledge, several new strategies for treating neurodegenerative diseases can be envisaged.

## Introduction

Many neurodegenerative diseases are characterized by an early loss of neurites, but little is known about the responsible mechanisms in the human central nervous system (CNS) [1–7]. An improved mechanistic understanding of the underlying pathophysiological processes may lead to new diagnostic and therapeutic strategies. The failure of various well-established anti-apoptotic strategies to prevent neurite degeneration [8–11] suggests that novel death mechanisms may play a pivotal role in neurites. Additional evidence for this hypothesis was provided by the discovery of an axon degeneration program in neurites of the peripheral nervous system (PNS) [12].

Sensory neurons of the PNS undergo a specific degeneration process upon mechanical injury or toxicant exposure (e.g. during tumor chemotherapy) [12]. A well-established model to study this is the cut-injury of nerves. This axotomy, leading to a degeneration of the distal part of the neurite, is termed “Wallerian degeneration” (WLD) [13]. WLD has been assumed to be a passive process, until the discovery of a mutant mouse with highly resilient axons. In this strain, called “Wallerian degeneration slow (WLD(s))” [14–17], the axonal synthesis of nicotinamide adenine dinucleotide (NAD^+^) via nicotinamide mononucleotide adenylyltransferase (NMNAT) enzymes is maintained for much longer time than in wild type (WT) mice. The reason is that WLD(s) mice express a protein in which the NAD^+^ synthesis protein NMNAT1 is fused to a small N-terminal fragment of ubiquitination factor E4B (Ube4b) [14]. This WLD(s) protein takes over the function of axonal NMNAT2. The latter is rapidly lost after axotomy [18]. The stabilization of NMNAT activity and NAD^+^ levels in WLD(s) mice increases axonal NAD^+^ levels and therefore greatly prolongs the axon’s life [19]. Thus, NAD^+^ levels are considered a cell-intrinsic sensor or pivot point for life-death decision within the axonal compartment [20]. This idea is further supported by the observation that NAD+ levels substantially decline in aging brain tissue [21].

The recent finding that the NAD^+^-degrading enzyme SARM1 [22–25] is involved in axon degeneration suggests that Wallerian degeneration is a programmed process [12]. The core of this hypothesis is that SARM1 activation occurs as a consequence of metabolite changes that follow the loss of NMNAT2 after axotomy. The axonal metabolome changes not only affect the NMNAT product NAD^+^, but also include an accumulation of the substrate nicotinamide mononucleotide (NMN) [26]. NMN activates SARM1 [24, 27], whereas NAD^+^ allosterically inhibits SARM1 [28, 29]. The decrease of NAD^+^, accompanied by increased NMN in injured neurites, controls SARM1 activation [30]. Activated SARM1 further degrades local NAD^+^ pools [23]. It remains unclear what exactly causes ND downstream of SARM1 activation, but a severe depletion of NAD^+^ leads to a breakdown of several metabolic pathways accompanied by the disturbance of calcium homeostasis [24, 31].

SARM1 can also be activated by exposure to the rodenticide Vacor [32, 33]. Vacor mononucleotide (VMN), generated by the nicotinamide phosphoribosyl transferase (NAMPT), acts as a strong activator of SARM1. Vacor toxicity to rodent peripheral neurons is completely abolished by SARM1 deletion [33, 34].

The role of SARM1 in rodent PNS neurons is clearly established. Also in human PNS neurons, WLD(s) expression protected against axotomy-induced neurite degeneration [35]; SARM1- dependent axonal degeneration was described in human iPSC-derived peripheral neurons [36, 37].

The evidence regarding programmed neurite death in the human CNS is rather sparse; and most data are preliminary [38, 39]. Data from mouse CNS models indicate (i) a protective effect of SARM1 knockout on optic nerve degeneration [40], (ii) attenuation of traumatic brain injury in SARM1 knockout animals [41, 42], (iii) protective effects of WLD(s) expression in some models of Parkinson’s disease [43, 44], (iv) protection of hippocampal or cortical neuron cultures by deletion of SARM1 [25, 38, 45].

For biomedical applications, the question is: “Does SARM1-play a role in the degeneration of neurites projecting from human CNS neurons?” [12, 46, 47]. The question is difficult, because not many human cell models of Wallerian-like degeneration have been established. Recently, cultures of fully differentiated, post-mitotic LUHMES neurons have been used to develop a specific neurite degeneration model that allows biochemical analysis in the neurite compartment [48]. Before this, such cells (dopaminergic midbrain neurons) have been used for 20 years as a model to study neurite degeneration and impaired neurite formation of human CNS neurons [49–52]. In the new setup, LUHMES-based spheroids can generate neurites of several mm length. They are suitable for axotomy studies due to the ease of inflicting a cut injury and because the degeneration process can be quantified exactly for individual neurites or larger populations [48].

In this novel model, we found previously that neurite disintegration was slowed by nicotinamide supplementation [48]. In the current study, we build on these pilot experiments to study the role of SARM1 in axotomy- and toxicant-induced neurite degeneration in human CNS neurons.

## Materials and methods

### Chemicals and media supplements

A list of all used chemicals and media supplements can be found in the supplementary material.

### Cell culture

LUHMES cells were cultured as previously described in detail [48, 53, 54] Briefly, proliferating LUHMES cells were cultured in poly-L-ornithine (PLO)/fibronectin (Fbn) coated T75 flasks (Sarstedt, Germany) in advanced DMEM/F12 supplemented with 1x N2, 2 mM Glutamine, and 40 ng/ml bFGF. Cells were passaged every 2-3 days. To passage cells, cells were detached with 0.05% Trypsin/EDTA (Gibco, USA). After detachment, suspended cells were diluted in advanced DMEM/F12 and centrifuged for 5 min at 300 x g. The supernatant was removed and cells were resuspended in the respective medium. Proliferating cells were seeded at a density of 40 000 cells/cm². To initiate differentiation, cells were seeded into T75 flasks in differentiation medium (advanced DMEM/F12 supplemented with 2 ng/ml GDNF, 2.25 μM tetracycline and 1 mM dibutyryl-cAMP) at a density of 135 000 cells/cm². LUHMES were differentiated for 2 days before they were seeded in either round-bottom plates for spheroid generation, or flat bottom 96-well plates for 2D neurotoxicity assessments (day of differention (DoD) 2). The LUHMES cell line (ATCC: CRL-2927) was genetically characterized in [55]. Annotated variant files for the LUHMES cell line (UKN Subpopulation) has been deposited for public access at ELIXIR Luxemburg (accession number: 10.17881/LCSB.20180321.01). Cells were regularly tested for mycoplasma contamination by Venor GeM Classic kit (Minerva Biolabs, Berlin, Germany).

### Spheroid generation, plating and neurite isolation

Spheroids were generated and handled as described in [48]. Briefly, DoD2 LUHMES cells were seeded into round-bottom plates (Corning 3795, USA) coated with FaCellitate BioFlex coating solution (2 min incubation at room temperature (RT)) at a density of 10 000 cells per well. Plates were centrifuged for 5 min at 300 x g. Spheroids were plated in 96-well flat bottom plates in Matrigel suspension (2.5%) in differentiation medium on DoD9. To this end, Matrigel was diluted in cold differentiation medium (5% V/V). Each well was coated with 50 µl Matrigel solution and incubated for 30 min at 37 °C. Spheroids were transferred in 50 µl into Matrigel suspension to reach a total volume of 100 µl. Spheroids were cultured for neurite outgrowth on Matrigel for 5 or 6 days. Axotomy was induced and neurites were isolated by removeing the centrally organized cell bodies with a pipette tip under visual control (as described in [48]).

### Western blots

Protein samples were generated and processed as described in [48]. Briefly, 5–10 wells of a 96 well plate of isolated neurites or whole cells were lysed in 10 µl Laemmli buffer per well and pooled subsequently. To analysis the cleavage of spectrin, samples were taken from isolated neurites in the presence of a pan-protease inhibitor cocktail (Roche, Switzerland). Lysates were heated for 5 min to 95 °C and were centrifuged for 1 min at 10 000× g through NucleoSpin Filters (Macherey-Nagel GmbH, Duren, Germany). Twenty microliters of lysates were loaded onto SDS (8-15%, depending on protein size) gels and until bromophenol blue bands reached the bottom of the gel. Proteins were transferred onto nitrocellulose membranes (Amersham, UK) using the iBlot 2 dry blotting system (Invitrogen, Waltham, MA, USA). Membranes were blocked with 5% (w/v) bovine serum albumin, or milk, in Tris buffered saline (TBS)-Tween (0.5% (V/V)) over night at 4 °C. Respective primary antibodies were incubated at 4 °C over night. Membranes were washed three times in TBS-Tween (0.5% (v/v)) at RT for 10 min, and then incubated with horseradish peroxidase-conjugated secondary antibodies for 1 h at RT. For visualization, ECL Western blotting substrate (Pierce/Thermo Fisher Scientific, Rockford, IL, USA) was used and chemiluminesence was recorded with a Fusion-SL 3500 WL device with Fusion software (Version 15.18, Bio-Rad, Hercules, CA, USA). Densitometric quantification was performed with an in-house software. The integrated intensity of pixels of a given protein was quantified. Background correction was performed by a single region with unspecific background signal. A list of used antibodies and image of full length western blots can be found in the supplementary material.

### Immunocytochemistry and fluorescence imaging

Immunocytochemistry and fluorescence microscopy was performed as described in [48]. Briefly, spheroids were plated in chambered polymer coverslips (ibidi 80826, Gräfelfing, Germany). Isolated neurites were fixed by replacing half medium with 10% neutral buffered formalin (Leica Biosystems Richmond, Inc., Richmond, IL, USA) and incubated for 30 min at RT. Samples were washed once with PBS (Gibco, USA) and blocked and permeabilized for 1 h at room temperature in blocking buffer (5% FBS, 0,1% Triton-X100 in PBS). Samples were incubated with primary antibodies diluted in blocking buffer at 4 °C over night. They were washed twice with blocking buffer. Secondary antibodies were diluted in blocking buffer and incubated for 1 h at room temperature. Samples were washed twice with PBS. Samples were covered with Aqua-Poly/Mount (Polyscience, Warrington, PA, USA) and left for drying until imaging. Images were recorded at an AxioObserver epifluorescence microscope (Zeiss, Oberkochen, Germany).

### Scanning electron microscopy

For scanning electron microscopy (SEM), spheroids were grown on minimal Matrigel coating. To this end, glass cover slips were pre-coated in 24-well plates with 86 µg/ml PLO over night at 37 °C. Subsequently, cover slips were incubated with 5% Matrigel in advanced DMEM/F12 for 30 min at 37 °C. The Matrigel suspension was replaced by 450 µl warm differentiation medium and spheroids were transferred in 50 µl onto Matrigel coating. Spheroids were left to grow out neurites for 5 days. Samples were fixed, processed and imaged as described previously [48].

### Measurement and quantification of degeneration

Degeneration of neurites was quantified as described in [48]. Briefly, neurites were stained with 2 µM calcein-AM for 30 min before imaging. Per condition, ten images of five technical replicates were recorded with an AxioObserver epifluorescence microscope (Zeiss, Germany). Images were binarized and objects were analyzed in FIJI (Version 1.57q). Neurite integrity was derived from the average size of objects in the image; fragmentation was derived from the total count of objects. Integrity was normalized to intact neurites (uncut or freshly isolated); fragmentation was normalized to completely fragmented, untreated wild type neurites (18 or 24 h after axotomy).

### Quantitative analysis of mitochondria

To evaluate live mitochondria in neurites, neurites were stained with 1 µM TMRE dilution in cell culture medium. Images were recorded an AxioObserver epifluorescence microscope (Zeiss, Germany). Obtained images were analyzed using FIJI (version 1.51q). Images were background corrected (subtract background pixel radius = 50 px), an unsharp mask (strength = 0.6, radius = 2 px) and a median filter were applied (radius = 1 px). The processed images were then automatically thresholded (method = “Otsu”) to binary images. A watershed filter was used to separate overlapping objects. Objects with a size of >1 µm² were analyzed. The number of mitochondria was normalized to the neurite covered area obtained by parallel Calcein-AM staining.

To analyze mitochondrial shape, neurites were immunostained and imaged with a 63x objective (plan-apochromat oil, NA = 1.4). Images were processed as described for TMRE stained mitochondria. To evaluate the shape, the aspect ratio (AR) of the mitochondria was used. Mitochondria with an AR > 3 were regarded as elongated, mitochondria with an AR < 1.5 were regarded as round.

### 2D neurotoxicity assessment

To asses neurotoxicity in LUHMES cells cultures in 2D, DoD2 LUHMES cells were seeded in PLO/Fbn coated 96-well plates (Sarstedt, Germany) at a density of 155 000 cells/cm² in differentiation medium. On DoD5, cells were treated 10 µl of a 10-fold concentrated toxicant solution. On DoD6, cells were stained with Calcein-AM (1 µM) and H-33342 (1 µM). Cells were imaged at the Cellomics Array-Scan VTI HCS Reader (ThermoFisher, PA, USA) as previously described [50, 56, 57]. Briefly, viability was assessed by the ratio of calcein/H-33342 double-positive cells vs. cells only stained by H-33342. Neurite area was assessed by measuring the area covered by calcein positive structures which were detected outside of cell somata. Cell somata were defined by expanding the outlines of detected nuclei by 3.2 µm. Viability and neurite area were normalized to untreated controls.

### Seahorse assay

In order to measure oxygen consumption of isolated neurites, 24-well Seahorse plates (Agilent, CA, USA) were pre-coated with 200 µl PLO (86 µg/ml) and Fbn (1 µg/ml) in milliQ H_2_O and incubated over night at 37 °C. Coating solution was removed and differentiation medium with 5% Matrigel was added. Plates were incubated for 30 min at 37 °C. Spheroids were transferred to the seahorse plates on DoD9 (one spheroid/well). Neurites were isolated on DoD15. Oxygen consumption was measured in the Seahorse XFe24 Analyzer (Agilent, CA, USA). To assess the functionality of the respiratory chain, inhibitors were sequentially injected: [1] oligomycin (1 µM), [2] FCCP (1.5 µM), and [3] rotenone/antimycin A (both 0.5 µM).

### ATP Assay

Neurite ATP was measured with the CellTiter-Glo™ 2.0 assay (G9061, Promega, USA) according to manufacturer’s instructions. Briefly, CellTiterGlo assay mix was diluted at equal part with PBS containing 0.5% Triton-X100. After neurite isolation, 50 µl reaction mix was added to cell culture medium and incubated for 5 min at RT on a shaker. Then, 100 µl were transferred to a white assay 96 well plate. Luminescence was measured in a plate reader (Infinite M200Pro, Tecan, USA). Data was normalized to ATP levels of freshly isolated neurites. Control experiments showed that ATP levels were not immediately affected by axotomy (data not shown).

### NAD(H) assay

The total pool of NAD^+^ and NADH in neurites was measured with the NAD/NADH-Glo™ assay (G9241, Promega, USA) according to manufacturer’s instructions. Briefly, medium of isolated neurites was removed and replaced with 25 µl DPBS. To each well, 25 µl of the reaction mix were added. 40 µl were transferred to a white assay 96 well plate. A dilution of NAD^+^ was used for assay calibration (highest concentration: 500 nM). Luminescence was measured every 5 min for 1 h in a plate reader (Infinite M200Pro, Tecan, USA). The strongest signal which was still in the linear range was used for analysis. Data was normalized to NAD^+^ levels measured in freshly isolated neurites.

### Caspase activity assay

To measure caspase activity, neurites were separated from their cell bodies. Medium was removed and neurites were lysed in 25 µl per 96-well (lysis buffer: 25 mM HEPES, 5 mM MgCl_2_, 1 mM EGTA, 1 mM AEBSF, 0.1% Triton-X100). Neurites were incubated in lysis buffer for 7 min at 37 °C. 90 µl of assay buffer was added per well (assay buffer: 50 mM HEPES, 1% Sucrose, 0.1% CHAPS, 10 mM DTT, 50 µM AC-DEVD-Afc). 100 µl of the reaction mix were transferred to a black assay plate and incubated for 30 min at 37 °C before fluorescence measurement in a plate reader (excitation: 376 nm, emission: 482 nm, Infinite M200Pro, Tecan, USA). A blank value was subtracted from all values for background correction. Values were normalized to caspase activity of neurites isolated immediately before the assay.

### SARM1 silencing

Silencing of SARM1 was performed by delivering siRNA to LUHMES cells. Transfection was performed according to manufacturer’s instructions: 0.2% Lipofectamine RNAiMAX and 40 nM Silencer Select siRNAs (both ThermoFisher, Waltham, USA) were used as transfection reagents. The cells were either transfected with: Silencer™ Select Negative Control No. 1 siRNA (Cat. no. 4390843), Silencer™ Select GAPDH siRNA (Cat. no. 4390849), SARM1-A siRNA (ID: s23032), or SARM1-B siRNA (ID: s23031; both Cat. no. 4390771). Transfection mix contained 2% Lipofectamine and 400 nM siRNA (10-fold). To test silencing efficacy, DoD2 LUHMES cells were transfected in suspension and cultured in 2D at a density of 550 000 cells/well in 12 well plates (Sarstedt, Germany) coated with PLO/Fbn. Cells were cultured until DoD8.

For the generation of transfected spheroids, DoD2 LUHMES cells were transfected in suspension and were seeded in ULA plates at density of 10 000 cells/well. Cells were then centrifuged to facilitate spheroid formation. After 4 days (DoD6), spheroids were plated and transfection was repeated. Neurite isolation was performed 3 days after plating (DoD9).

### Transgenic LUHMES lines

WLD(s) expressing LUHMES were produced by lentiviral transduction and characterized previously in our group [58]. To generate LUHMES expressing dominant negative SARM1 (dnSARM1) [59], a lentiviral vector was created that uses a 4-hydroxytamoxifen inducible system [60]. The regulating gene Gev16 was fused to a selectable marker (BSD conferring Blasticidin S resistance) via a 2 AUbi cassette. The ribosomal skipping of the 2A sequence (Arber et al., 2013) will produce a minimally extended blasticidin S deaminase (BSD) protein that is fully functional. The translation will also produce a UbiGev16 fusion protein [61]. The latter will be cut by cellular hydrolases into an Ubi and a Gev16 protein. As this process is accurate on the amino acid, the Gev16 produced will be identical to the originally described Gev16.

The inducible system was integrated into a lentiviral vector whose synthetic 5xUA promoter system [60] drives a fusion of tRFP and dnSARM1.

Messenger RNA, isolated from LUHMES cells, was transcribed into cDNA that was used to create dnSarm1 via PCR. Oligonucleotides were designed with the AiO software [62] so the gene could be created from just three parts, introducing the necessary mutations (K193R, H194A and H685A; [59]) and extra sites for restriction enzymes at the same time. The parts were cloned via TOPO cloning (Invitrogen), sequenced and assembled.

Viruses were produced in HEK293FT cells (Invitrogen, USA); the supernatant was filtered and concentrated 100-fold using Amicon Ultra-15 centrifugal filters (Sigma-Aldrich, USA).

Proliferating LUHMES cells were infected and subsequently selected for 10 days under 2 µg/ml Blasticidin S. Single colonies were picked manually, expanded and tested for expression of both tRFP (by microscopy) and dnSARM1 (by Western blotting). Colonies were selected for universality of tRFP expression and high inducibility of dnSARM1. One clone whose characteristics corresponded optimally with these criteria was used in all further experiments.

### Statistical analysis

Except stated otherwise, displayed data are the means of (±SD or SEM) of biological replicates (different cell preparations). Each biological replicate was calculated as the means of at least 3-5 technical replicates (different wells or neurite preparation within the same experiment). Sample size of technical replicates was chosen to cover intra-experimental variance. Experiments were repeated in three independent biological replicates, except stated otherwise. Acceptance criteria for neurite samples were defined in [48]. Statistics was applied to the biological replicates only (conservative approach). Statistical analysis was performed with GraphPad Prism (V8.0.2). Statistical tests and significance levels are indicated in the figure legends. *p*-values < 0.05 were regarded as statistically significant. Statistical hypothesis testing was used to correct for multiple comparisons (see figure legends).

## Results and discussion

### NAD^+^ metabolism and axotomy-induced neurodegeneration (AIND)

**Fig. 1:**
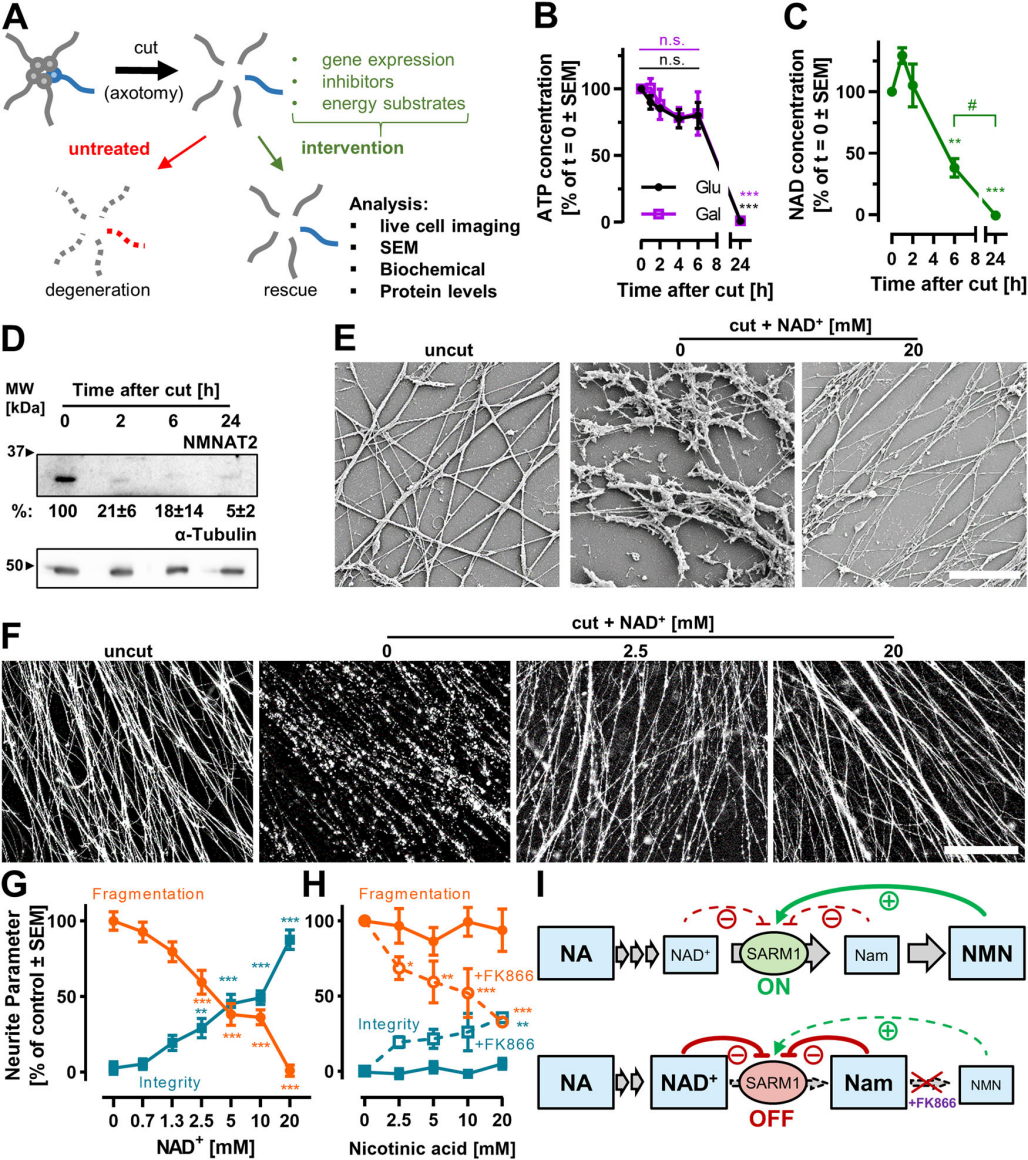
Axotomy- induced neurite degeneration is affected by NAD^+^ metabolism. **A** LUHMES neurons (human CNS) were cultured as attached spheroids with a corona of neurites (2.5D setup). Axotomy was performed by physical removal of the cell bodies. Neurite cultures were analyzed at different time points after the axotomy in presence or absence of metabolic modifiers. **B** ATP was measured in isolated neurites at different time points after axotomy. Cells were either cultured in standard medium (glucose containing), or in medium containing galactose instead of glucose. Control experiments showed that neurites represented about 35% of the whole cell culture mass (protein levels) and that ATP levels immediately before and after the cut were similar. **C** The change of total NAD^+^/NADH (NAD) was measured in isolated neurites after axotomy. Data are means from biological triplicates with ≥5 technical replicates. Significance was evaluated by ANOVA relative to 0 h. **p < 0.01, ***p < 0.001; ^#^*p* < 0.01 between time points. **D** NMNAT2 protein levels were analyzed by western blots. Average protein levels are indicated in %±SD of control (0 h), *N* = 3. **E**, **F** Plated spheroids were treated with different NAD^+^ concentrations immediately before axotomy. Neurites were fixed at 18 h after axotomy. **E** Images were recorded by scanning electron microscopy. Scale bar = 10 µm. **F** Neurites were stained with calcein-AM at 18 h after axotomy and imaged by epifluorescence microscopy. Scale bar = 200 µm. **G**, **H** Neurite integrity and fragmentation were quantified 18 h after cut in the presence of (**G**) different NAD^+^ concentrations. **H** Cells were treated with nicotinic acid (NA) alone or in combination with 500 nM FK866 (dotted line). Data are means from biological triplicates with 5 technical replicates each. Significance was evaluated by ANOVA followed Dunnet’s post hoc test (relative to solvent control). *p < 0.05, **p < 0.01, ***p < 0.001. **I** Schematic model of the synergistic effect of nicotinic acid (NA) and FK866 on SARM1 activity. Gray arrows indicate metabolic conversion; green/red arrows indicate activation/inhibition. Box sizes indicate levels of the metabolites. NMNAT2 is assumed to be depleted after axotomy so that NMN does not react to NAD^+^. In absence of FK866, nicotinic acid mononucleotide (NMN) activates SARM1, which leads to NAD^+^ depletion. NA cannot counteract this depletion. When NMN synthesis is blocked by FK866, then nicotinamide (Nam) accumulates and inhibits SARM1. In this situation, NAD^+^ levels, supported by NA supplementation, remain high. See Fig. S1 for more details.

To study the metabolic regulation of neurite degeneration (ND) in the CNS, we chose axotomy as the damage model to avoid the effects of chemicals that directly disrupt metabolism. (Fig. S1A). In our setup, the separation of cell bodies and neurites allowed the biochemical analysis of the neurite compartment [48] and allowed several interventions (Fig. 1A).

In line with our observation that severed neurites remained structurally intact for at least 6 h [48], ATP levels remained high for >6 h (independent of glycolysis substrates) (Fig. 1B).

NAD^+^ levels declined faster, so that >50% were lost within 6 h (Fig. 1C). This was in concordance with the fast degradation of the main axonal NAD^+^-synthesis enzyme, NMNAT2, within 2 h (Fig. 1D). Neurites supplied with external NAD^+^ remained structurally intact for at least 18 h after cut (Fig. 1E, F); thus, NAD^+^ strongly delayed axotomy-induced neurodegeneration (AIND). Upon loss of NMNAT2, nicotinamide mononucleotide (NMN) has been found to accumulate [26]. The shifted ratio of NAD^+^/NMN activates SARM1 [30] (Fig. S1B). To test this model of metabolic regulation in human CNS neurons, we first treated neurites with the NAD^+^ precursor nicotinic acid (NA) alone. As expected, it was not protective. Neither was a block of NAMPT, the NMN synthesizing enzyme. However, co-treatment with the NAMPT inhibitor FK866 and NA strongly delayed AIND (Fig. 1H, I). These results indicate a central role of NAD^+^ metabolism in AIND of human CNS neurons. The regulatory role of various NAD^+^-related metabolites was further supported by the finding that nicotinamide (Nam) protected neurites, and that the addition of FK866 did not change this robust protection (Fig. S1C).

### Mitochondrial function following AIND

**Fig. 2:**
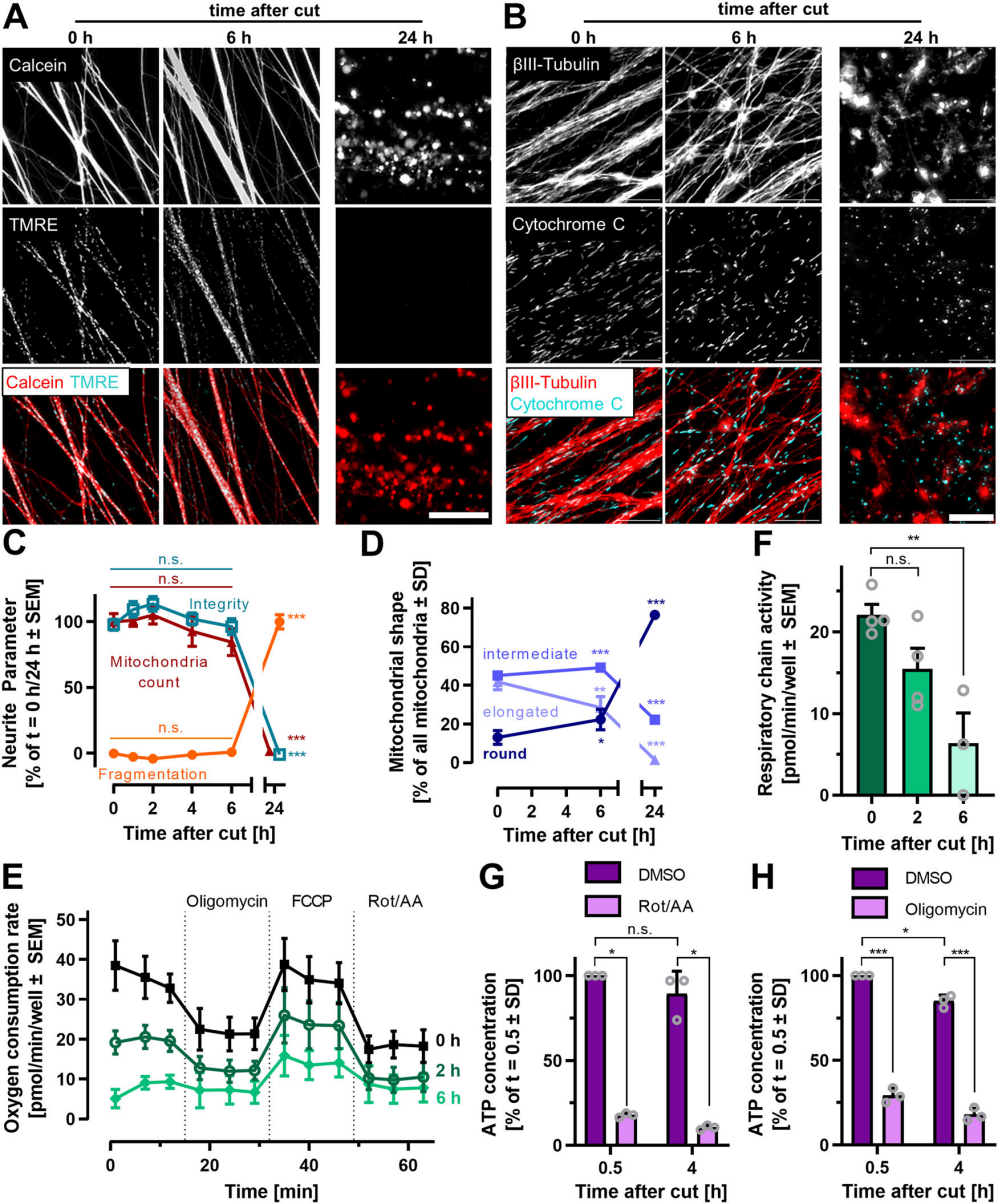
The role of mitochondria in acute axotomy-induced neurite degeneration. **A**, **C** Isolated neurites were stained with calcein-AM (3.2 µM) and TMRE (1 µM) at different time points after axotomy and imaged by epifluorescence microscopy. 0 h = few minutes after axotomy. Scale bar = 25 µm. Neurite integrity, fragmentation and number of TMRE^+^ mitochondria (mitochondria count) were quantified. Data are from biological triplicates with 5 technical replicates. Significance was evaluated by ANOVA/Dunnet’s post hoc test. n.s. = not significant. ***p < 0.001. **B**, **D** Isolated neurites were fixed and immunostained at different time points after axotomy and imaged by epifluorescence microscopy. Scale bar = 10 µm. Mitochondria (cytochrome C stain) with a length/width ratio <1.5 were classified as “round”, a ratio > 3 was classified as elongated. The shape of >100 mitochondria was evaluated in 5 independent fields. *p < 0.05, **p < 0.01, ***p < 0.001. **E**, **F** Different mitochondrial functional states were generated by sequential addition of oligomycin (blocked ATP-synthase), FCCP (maximal respiration by uncoupling) and rotenone/antimycin A (Rot/AA; blocked electron transport chain). OCR were measured in isolated neurites at 6 h, 2 h or a few minutes (0 h) after axotomy in 3–4 cell preparations. For each of these experiments, 3–5 technical replicates were measured. **F** Data indicate the respiratory chain activity, i.e. the oxygen consumption rates (OCR) without any inhibitor, corrected for the OCR after inhibitor addition. ***p < 0.001 by ANOVA with Dunnet’s post hoc test. **G/H:** Plated spheroids (2.5D cultures) were treated with GluTor (100 nM) for 24 h before axotomy to block glycolytic activity. The ATP content was measured 30 min or 4 h after axotomy. The experiment was performed in presence or absence of mitochondrial inhibitors (**G**: rotenone/antimycin A (Rot/AA); H: oligomycin), added 30 min before sample preparation (lysis). Data are means from biological replicates. Significance was evaluated by two-way ANOVA followed by Tukey’s post hoc test. n.s. = not significant, *p < 0.05.

Because of the close connection of NAD^+^ synthesis with cellular energy metabolism, we explored how mitochondria were affected by AIND. In concordance with the relatively stable ATP levels in the initial hours after axotomy, mitochondrial membranes remained polarized (accumulation of TMRE) for at least 6 h (Fig. 2A, C). As neither the rate of AIND, nor the rate of mitochondrial depolarization changed when glucose was removed from the medium, we suggested that ATP levels were maintained during this time mainly by continuously working mitochondria (Fig. 1B, S2A, B). Consistent with this, cytochrome c remained localized to mitochondria. The mitochondrial morphology changed from a rather elongated shape to a more roundish shape within 6 h after axotomy. Clear swelling and fragmentation was only observed thereafter (Fig. 2B, D). The morphology, together with the TMRE data (Fig. S2A, B) suggest an intact mitochondrial inner membrane (Fig. 2B, D). However, mitochondrial membrane polarization may be maintained either by active respiratory chain activity, or by a reversal of the proton pump (using up ATP). To distinguish these effects, we measured the oxygen consumption of neurites. Basal oxygen consumption was 22 ± 1 pmol/min/well in isolated neurites (i.e. within the range expected for the viable non-cut neurite compartment). This dropped to 15 ± 3 pmol/min/well after 2 h and 6 ± 4 pmol/min/well after 6 h (still >25% of the full activity) (Fig. 2F). Even after 6 h, the remaining, mitochondrial oxygen consumption could still be blocked by the specific F_1_/F_0_ ATPase inhibitor oligomycin, and the consumption was largely increased by the mitochondrial uncoupler FCCP. Together, this points to a functional respiratory chain that declined in activity, but was still coupled in a sizeable mitochondrial fraction after 6 h (Fig. 2E).

To test the role of mitochondria in the continuous replenishment of neurite ATP pools, we followed ATP levels after a block of the respiratory chain at different times relative to axotomy. Glycolysis was disabled in these experiments (by the block of glucose uptake, using Glutor). ATP levels dropped within minutes after block of the respiratory chain (Fig. 2G) or after block of the mitochondrial ATPase (Fig. 2H). This indicates (i) a fast turnover of ATP in isolated neurites and (ii) an ongoing ATP synthesis by oxidative phosphorylation for at least 4 h after axotomy. In the presence of glucose, neurites could fully compensate respiratory chain inhibition, which suggests the ability of neurites to switch to ATP generation by glycolysis, when required.

We conclude that some functional capacity of mitochondria was maintained for at least 6 h after axotomy. Our data suggests that ATP was continuously used for metabolic processes and replenished by ongoing re-synthesis in isolated neurites. Thus, NAD^+^ decline (fast) and ATP loss (slow) do not seem to be directly coupled. The rapid depletion of NAD^+^ appears to be rather a direct consequence of axotomy than an indirect consequence of failing cellular energy metabolism.

### Caspase independence of AIND

**Fig. 3:**
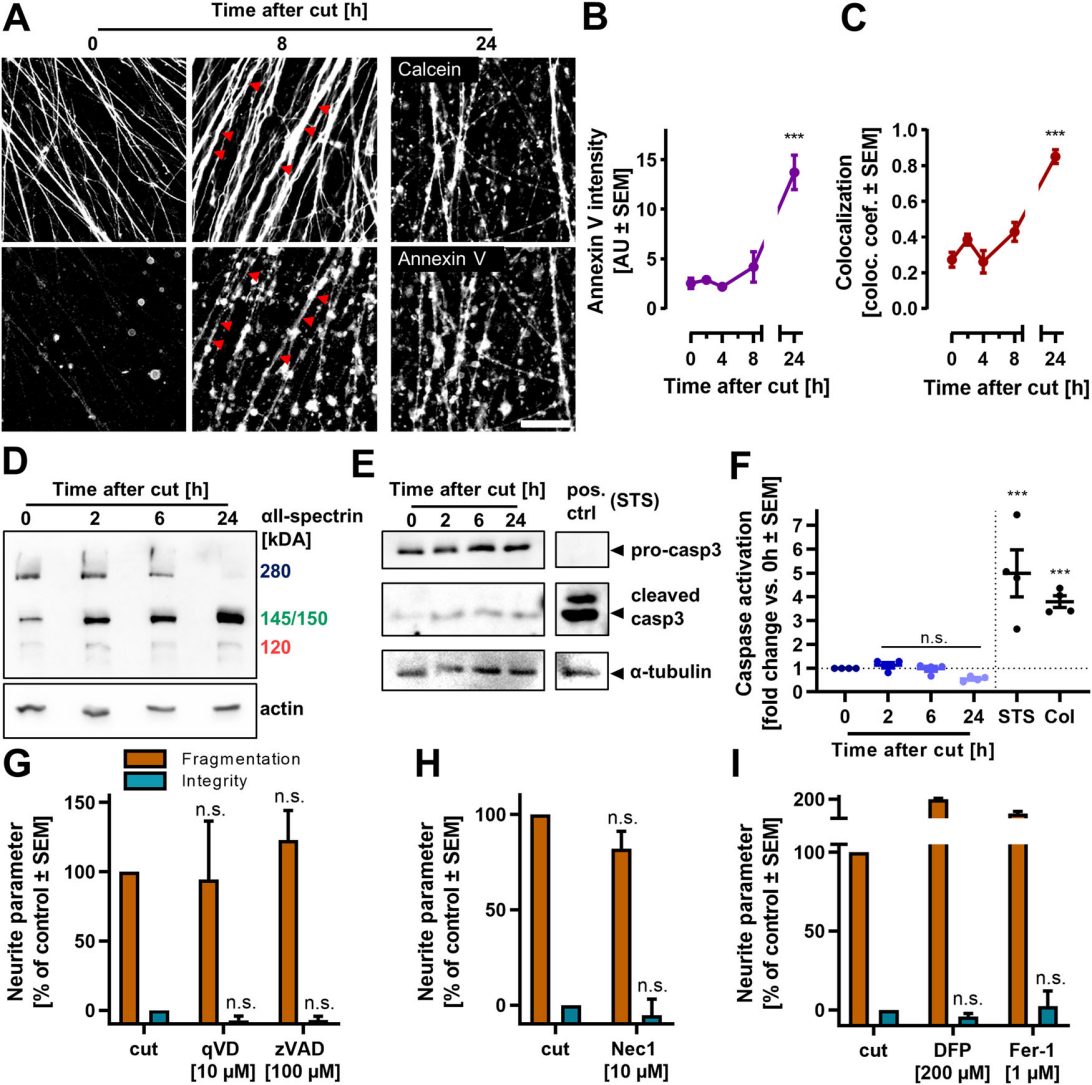
Axotomy-induced neurite degeneration in the absence of caspase activation. **A** Neurites were cut, and stained with orange calcein-AM and FITC-annexin V at different time points after axotomy. Images were recorded by epifluorescence microscopy and show the same field of view in different fluorescent channels. Arrowheads indicate two exemplary double-positive neurites at 8 h after axotomy. Scale bar = 50 µm. **B**, **C** The integrated intensity (**B**) of the annexin V signal was evaluated from fluorescent microscopy images, and (**C**) the annexin V colocalization with calcein positive structures was quantified. Mander’s colocalization coefficient (1 = total colocalization, 0 = no colocalization) was calculated by ImageJ’s colocalization threshold function. ***p < 0.001 by ANOVA with Dunnet’s post hoc test. **D** Western blot analysis of α-spectrin ( = fodrin) in isolated neurite protein at different time points after axotomy. The blot is representative of three blots from biological replicates (cf. Fig S3A). **E** Western blot analysis of pro-caspase3 (pro-casp3) and cleaved caspase3 of in the protein fraction from isolated neurites at different time points after axotomy. LUHMES cells, treated with 100 nM staurosporine (STS) for 24 h on DoD6, were used as positive control. The blot is representative of three blots from biological replicates (cf. Fig S3B). **F** Caspase activity was measured in isolated neurites at different time points after axotomy. 2.5D LUHMES cultures (see Fig. S1), treated with either 100 nM staurosporine (STS) or 100 nM colchicine (Col) for 24 h were used as positive controls. Cell bodies of these positive controls were removed immediately before cell lysis. Thus, caspase activity was measured only in the neurite compartment. n.s. = not significant, ***p < 0.001 by ANOVA with Dunnet’s post hoc test. **G**–**I** Neurite degeneration was quantified in axotomized neurites in the presence of cell death inhibitors. 2.5D LUHMES were treated with inhibitors of caspases (**G**), necroptosis (**H**) or ferroptosis (**I**) 30 min before axotomy. At 18 h after axotomy, neurites were stained with calcein and imaged. qVD = quinoline-Val-Asp-difluorophenoxymethylketone, zVAD = carbobenzoxy-valyl-alanyl-aspartyl-[O-methyl]-fluoromethylketone, Nec1 = necrostatin, DFP = deferiprone, Fer-1 = ferrostatin. n.s. = not significant, *p < 0.05, ***p < 0.001 by ANOVA with Dunnet’s post hoc test.

The morphology of the AIND resembled somehow the membrane blebbing typically observed during apoptotic cell death. To test whether apoptotic processes were involved in AIND, we stained phosphatidyl serine (PS) with fluorescently labeled annexin V. Cut neurites began to expose PS at 8 h after axotomy. This staining increased at later stages of degeneration (Fig. 3A, B). This increase was not mainly a consequence of membrane rupture, as annexin V positive structures co-localized with calcein-stained structures (Fig. 3C). The exposure of PS and maintenance of membrane integrity are typical features of apoptosis. To further elucidate the involvement of apoptosis in AIND, we investigated the cleavage of fodrin (αII-spectrin) in axotomized neurites. Full length fodrin appeared to decline as early as 2 h after axotomy. 24 h after axotomy, full length fodrin was undetectable (Fig. 3D, Fig. S3A). Analysis of the fragments revealed no increase in the caspase specific fragment (120 kDa), but an increase of the non-caspase fragment (150 kDa). The absence of cleaved caspase 3 (Fig. 3E, Fig S3B) and the absence of caspase activity (Fig. 3F) in cut neurites confirmed AIND-induced fragmentation to be caspase-independent. Note that caspase activity was detectable in isolated neurites when known apoptotic triggers were used (staurosporine or colchicine, Fig. 3F).

Accordingly, AIND was not prevented by pan-caspase inhibitors (Fig. 3G). Inhibitors of other modes of cell death, such as necroptosis (Fig. 3H) or ferroptosis (Fig. 3I) also failed to protect from AIND. In conclusion, AIND showed some morphological features of apoptosis (PS exposure, maintained membrane integrity), but was caspase-independent and it was not delayed by intervention with other cell death pathways. The increase of the 150 kDa fodrin fragment points towards the involvement of other proteases. However, calpain inhibition failed to prevent AIND in this system previously [48].

### Pharmacological inhibition of SARM1 protects from AIND

**Fig. 4:**
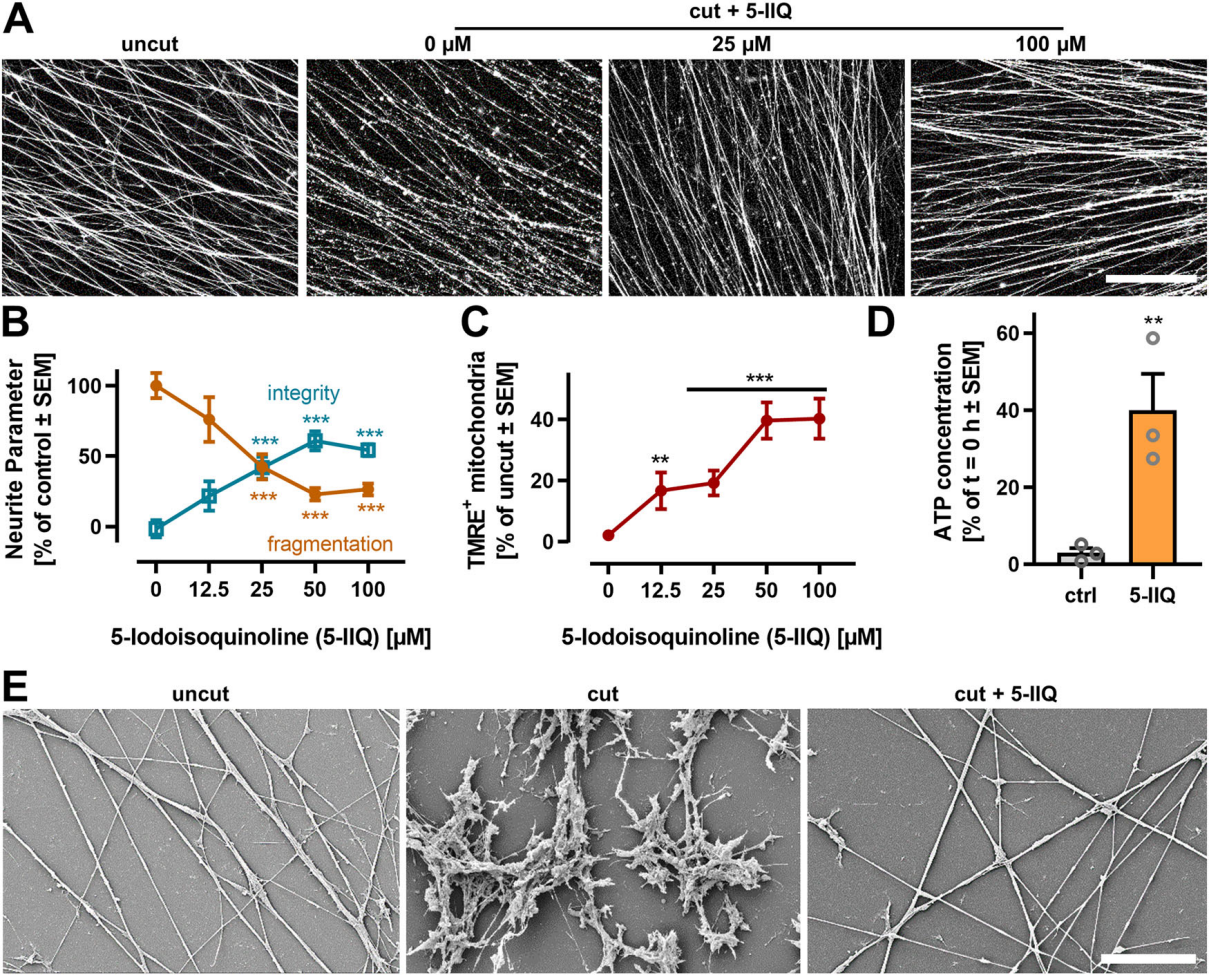
Protection of neurites from AIND by pharmacological inhibition of SARM1. 2.5D LUHMES were treated with different 5-iodoisoquinoline (5-IIQ) concentrations 30 min before axotomy. Neurites were analyzed 18 h after axotomy. **A** Neurites were stained with calcein-AM and TMRE and imaged by epifluorescence microscopy 18 h after axotomy. Representative images of calcein stained neurites are shown. Scale bar = 200 µm. **B** Neurite fragmentation and integrity were quantified by an image analysis algorithm at 18 h after cut. For each biological replicate, 10 fields (each covering 0.32 mm²) of 3-5 technical replicates of calcein stained neurites were recorded. Data is from three independent biological replicates. **C** The number of TMRE^+^ mitochondria was determined by an image quantification algorithm. Images were recorded as in (**B**). Data for each image were normalized to the neurite area in the image. In the end, data were normalized to uncut controls. **p < 0.01, ***p < 0.001 by ANOVA with Dunnet’s post hoc test. **D** ATP was measured after lysis of all neurites in a well. Neurites were treated with or without 50 µM 5-IIQ 30 min before axotomy. Data was normalized to ATP measured in freshly isolated neurites (*t* = 0 h). “Ctrl” refers to untreated neurites 18 h after axotomy. **p < 0.01 by Student’s *t* test. **E** Isolated neurites were obtained as above, but they were grown on glass coverslips with minimal Matrigel coating. Samples were fixed and processed before imaging by scanning electron microscopy (SEM). Representative images are shown. Scale bar = 10 µm.

The absence of classical cell death pathways suggests an alternative mode of neurite degeneration. SARM1 acts as a metabolic sensor of NAD^+^ levels in neurons and is responsible for AIND in the PNS. To test if SARM1 also mediates AIND in human CNS neurons, we inhibited SARM1 with 5-iodoisoquinoline [37, 63]. SARM1 inhibition prevented AIND in a concentration-dependent manner (Fig. 4A, B). The mitochondrial membrane potential was maintained in protected neurites for 18 h after axotomy (Fig. 4C). Isolated neurites, treated with 5-IIQ, also maintained ATP for 18 h (Fig. 4D), which suggests ongoing ATP synthesis. SEM imaging revealed that neurites remained structurally intact for 18 h after axotomy, when SARM1 was inhibited (Fig. 4E). These findings imply a central role of SARM1 in human CNS neurite degeneration and exemplify pharmacological SARM1 inhibition as a potential intervention.

### Sensitivity of human CNS neurons to Vacor toxicity mediated by SARM1

**Fig. 5:**
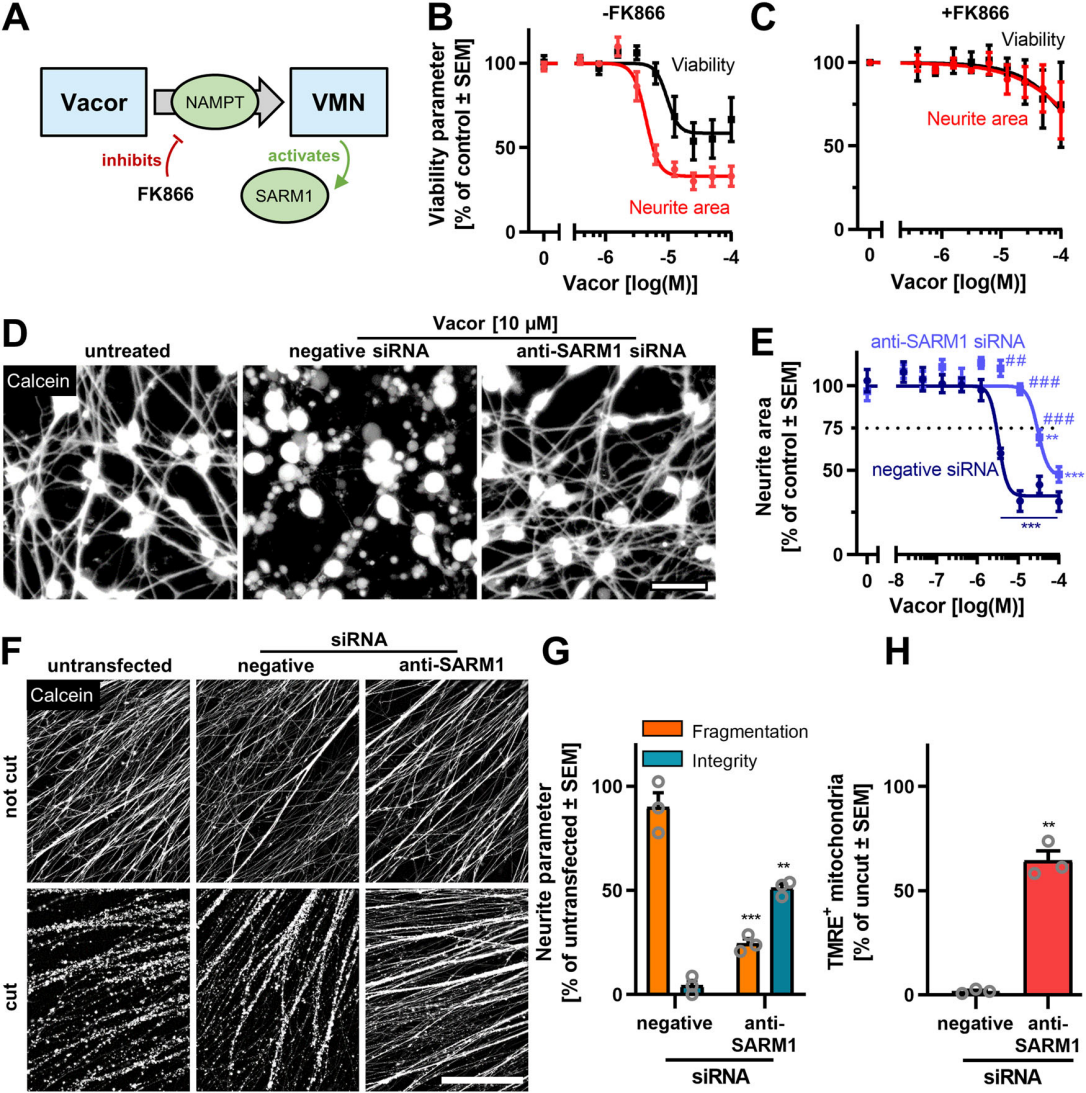
Knockdown of SARM1 protects from Vacor toxicity and AIND. **A** Schematic indicating how Vacor is metabolized intracellularly by NAMPT to the toxic metabolite Vacor mononucleotide (VMN), which acts as a strong SARM1 activator. NAMPT can be specifically inhibited by FK866. **B** LUHMES cells were seeded on DoD2 and were grown until DoD6. Cells were exposed to Vacor on DoD6 for 18 h. Cells were stained with calcein-AM and H-33342 and imaged by epifluorescence imaging. The neurite area and cell viability were quantified by an image analysis algorithm. Each data point is the means ± SEM of three biological replicates with three technical replicates each. Data was normalized to untreated controls. **C** Cells were treated and analyzed as described in (**B**), but were treated with 500 nM FK866 30 min before exposure to Vacor. **D** LUHMES cells were left untransfected or were transfected as cell suspensions with siRNA (negative control (Silencer Select non-targeted siRNA) or targeting SARM1 (Silencer Select; ID: s23031)) on DoD2. Then, cells were plated and grown until DoD6. These cells were exposed to Vacor for 18 h, or were left untreated. Cells were stained with calcein-AM before epifluorescence imaging (cf. Fig S4A). Representative images are shown. Scale bar = 50 µm. **E** Neurite area of LUHMES cells transfected with either negative siRNA, or siRNA targeting SARM1, was quantified after 18 h exposure to different concentrations of Vacor (note the logarithmic scaling). Significance between conditions was evaluated by ANOVA with Bonferroni’s post hoc test. ^##^*p* < 0.01, ^###^*p* < 0.001. Significance between treatments and control was evaluated by ANOVA with Dunnet’s post hoc test. **p < 0.01, ***p < 0.001. **F** LUHMES cells were transfected with siRNA on DoD2 (in suspension) and cultured for four days in low-adherence round-bottom plates to generate spheroids. These were transfected a second time and then plated. Axotomy was performed after 3 days of neurite outgrowth (on DoD9). Neurites were stained 18 h later with calcein-AM and TMRE and images were obtained by epifluorescence microscopy (cf. Fig. S5A). Scale bar = 200 µm. **G**, **H** Quantification of (**E**) neurite fragmentation and integrity and (**F**) number of TMRE^+^ mitochondria (as in Fig. 4C). Data were normalized to untransfected controls. **p < 0.01, ***p < 0.001 by ANOVA with Dunnet’s post hoc test.

SARM1 cannot only be activated by changes in NAD^+^ metabolism after axotomy, but also directly by chemicals. The rodenticide Vacor is intracellularly metabolized to VMN, which acts as a strong SARM1 activator [33] (Fig. 5A). We found that exposure to Vacor caused degeneration of LUHMES neurites. Neurite degeneration was observed at neurite-specific, non-cytotoxic concentrations (3 µM). This selectivity implies a specific role of SARM1 activation in neurite degeneration (Fig. 5B). The toxicity was mitigated when the conversion of Vacor to its toxic metabolite was prevented by the addition of FK866 (Fig. 5A, C). To test whether this effect was specifically caused by SARM1 activation, we knocked down SARM1 in LUHMES cells by transfecting them with siRNAs (Fig. S5C, D). Knockdown of SARM1 decreased the sensitivity of LUHMES cells against Vacor toxicity by a factor of ~10 (Figs. 5E, S5A, B). These suggest a role of SARM1 activation in human CNS neurites, and they provide evidence of knockdown efficiency for further studies.

### Mitigation of AIND through SARM1 knockdown or by expression of dominant negative SARM1 (dnSARM1)

To specifically check the role of SARM1 in AIND, we compared the extend of degeneration at different SARM1 expression levels (Fig. S5A–C). Knockdown of SARM1 prevented AIND (Fig. 5F, G). Neurites resisted degeneration for at least 18 h after axotomy and maintained the membrane potential of >60% of their mitochondria (Fig. 5H).

**Fig. 6:**
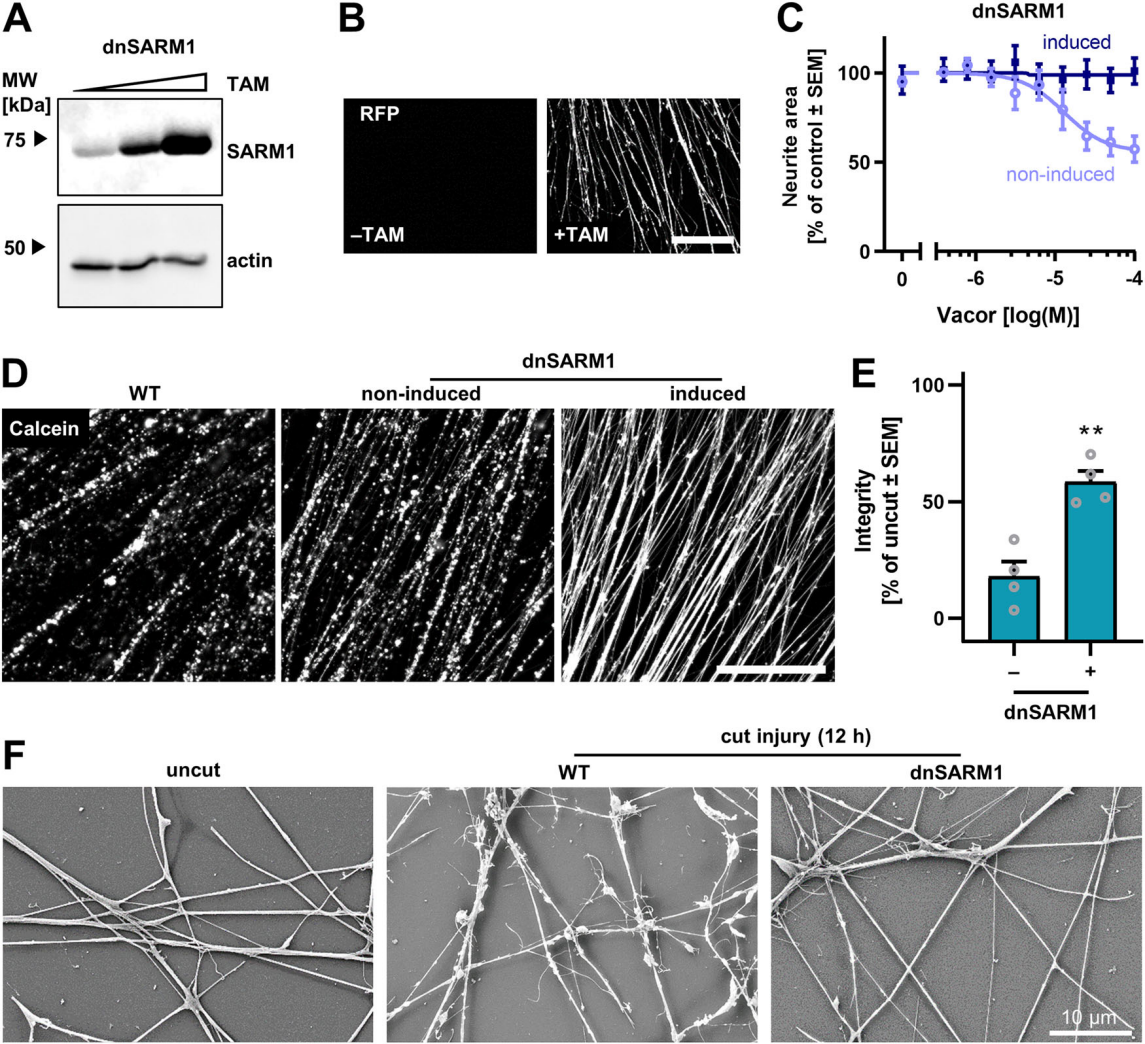
Expression of dominant negative SARM1 (dnSARM1) protects LUHMES from AIND and Vacor toxicity. LUHMES cell were lentivirally transduced to stably express an inducible construct controlling the expression of dominant negative SARM1 (dnSARM1) and turbo red fluorescent protein (tRFP). Because SARM1 functions as an octamer, certain point mutations in SARM1 disturb the formation and function of the SARM1 octamer. This mutated SARM1 isoform thereby acts as a dominant negative suppressor of endogenous SARM1 function [59]. **A** Expression of dnSARM1 was induced with tamoxifen (TAM) in plated spheroids of LUHMES cells for 6 days. On DoD15, axotomy was performed to obtain isolated neurites. Lysates were produced and analyzed by western blots for the total amount of SARM1 (endogenous SARM1 + dnSARM1) (cf. Fig S6B). The triangle symbolizes the increasing TAM concentrations used: 0 nM, 2 nM, 20 nM. **B** Inducibility of the construct by TAM was also tested as follows: On DoD15 (after exposure to TAM for 6 days), images of neurites were recorded by epifluorescence microscopy to monitor the expression of tRFP from the dnSARM1/tRFP construct (cf. Fig S6C). Scale bar = 200 µm. **C** LUHMES cells with the dnSARM1 construct were treated with Vacor on DoD6 for 18 h. Expression of dnSARM1 was induced with 0 nM or 20 nM TAM on DoD2. Cells were stained with calcein-AM and Hoechst-33342 and imaged by epifluorescence imaging. The neurite area and cell viability (Fig. S6D) were quantified. **D** The expression of dnSARM1 was induced in plated spheroids on DoD9. On DoD14, axotomy was performed. After 18 h, neurites were stained with calcein-AM and imaged by epifluorescence imaging. Representative images are shown. Scale bar = 200 µm. **E** Neurite integrity was quantified by an image analysis algorithm in neurites with or without dnSARM1 induction. Data was normalized to intact, uncut neurites. Images were obtained as described in (**D**). Each data point represents a biological replicate with 10 field recorded from 3-5 technical replicates. Neurite fragmentation is shown in Fig. S6D. **p < 0.01 by Student’s *t* test. **F** Isolated neurites were obtained as above, but they were grown on glass coverslips on minimal Matrigel coating. 12 h after axotomy, samples were fixed and processed before imaging by scanning electron microscopy. Representative images are shown.

An alternative intervention against SARM1-dependent neurite degeneration is the expression of dominant negative isoforms [59]. To test if this intervention is feasible in human CNS neurons, we transduced LUHMES cells with a tamoxifen (TAM)-inducible construct of dominant negative SARM1 (dnSARM1, Figs. 6A, S6A). The expression of dnSARM1 was monitored by the expression of red fluorescent protein (tRFP, Figs. 6B, S6C). Induction of dnSARM1 expression completely abolished Vacor toxicity (Fig. 6C). These control experiments demonstrated the suppression of WT SARM1 activity by dnSARM1 in LUHMES cells. Most importantly, neurites of dnSARM1 expressing cells were resistant to AIND, compared to non-induced and WT cells (Figs. 6D, E, S6D). SEM imaging revealed that rescued neurites did not show signs of degeneration for at least 12 h after axotomy (Fig. 6F). These results show that dnSARM1 also exerts its neurite-protective function in human dopaminergic neurons and further demonstrates that SARM1 function is essential for AIND.

### Stabilization of NAD^+^ levels by “Wallerian degeneration slow” (WLD(s)) expression

**Fig. 7:**
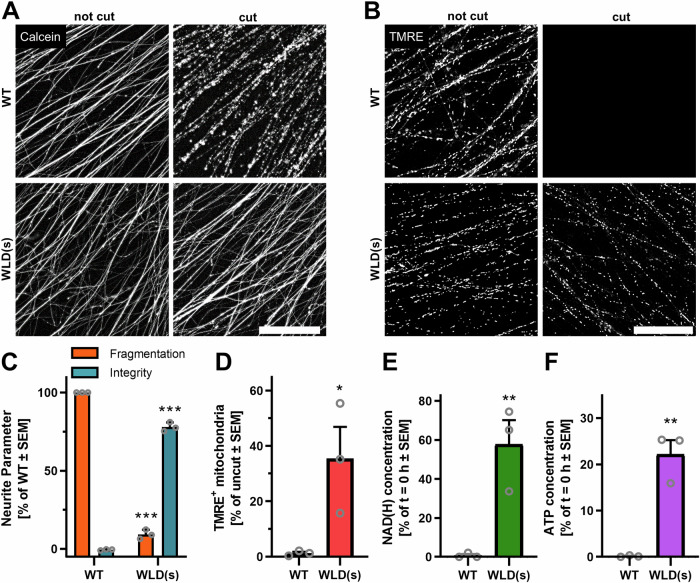
Expression of WLD(s) protects neurites from AIND. LUHMES cells were lentivirally transduced to express the “Wallerian degeneration slow” (WLD(s)) fusion protein [58]. Spheroids were generated from WT and WLD(s)-expressing cells. Spheroids were plated on DoD9. On DoD15, axotomy was induced and neurites were isolated. **A**, **B** Neurites of WT and WLD(s)-expressing LUHMES cells were left intact, or axotomy was induced. 18 h later, neurites were stained with (**A**) calcein-AM and (**B**) TMRE and were imaged by epifluorescence imaging. 10 fields (0.32 mm² each) each were recorded per condition in three biological replicates. Representative images are shown. (**A**) Scale bar = 200 µm, (**B**) scale bar = 50 µm. **C** Neurite integrity and fragmentation was evaluated from calcein stained cut neurites by an image analysis algorithm. Images were obtained as in (**A**). Data of WLD(s) neurites was normalized to neurite parameters of intact (100% integrity) WLD(s) neurites or fully degenerated (100% fragmentation) WT neurites. ***p < 0.001 by ANOVA with Dunnet’s post hoc test. **D** The number of TMRE^+^ mitochondria was quantified by an image analysis algorithm from images recorded as described in (**B**). Data from each image was normalized to the neurite area in each image. The relative number of TMRE^+^ mitochondria was normalized to respective intact controls. *p < 0.05 by Student’s *t* test. **E** The total pool of NAD^+^ and NADH (NAD(H)) was measured in WT or WLD(s) neurites after lysis of all neurites in a well. Data was normalized to NAD^+^ measured in freshly isolated neurites of the respective genotype (*t* = 0 h) (cf. Fig S7A). **p < 0.01 by Student’s *t* test. **F** ATP was measured and normalized analogously to the NAD(H) measurements described in (**E**) (cf. Fig S7B). **p < 0.01 by Student’s *t* test.

SARM1 activation is facilitated by a loss of NMNAT2 and the resulting disturbance in NAD^+^ metabolism. The fusion protein WLD(s) was found to replace the catalytic function of NMNAT2 in the axons of mice, thereby preventing AIND. Here, we tested if this metabolic intervention also is protective in human midbrain neurons. Expression of WLD(s) in LUHMES cells potently protected neurites from AIND (Fig. 7A, C). Mitochondria of rescued neurites maintained their membrane potential for at least 18 h after axotomy (Fig. 7B, D). Isolated WLD(s) neurites maintained about 60% of their initial NAD^+^ levels for 24 h after axotomy (Fig. 7E), and the early drop (6 h after axotomy) observed in WT neurites was mitigated (Fig. S7A). This stabilization of NAD^+^ levels suggests a functional NAD^+^ synthesis by WLD(s). The ATP levels of WLD(s) expressing neurites dropped to 20% of control at 24 h after axotomy (Figs. 7F, S7B). This drop indicates on one hand a significant functional loss; on the other hand, maintenance of 20% ATP is still in the 0.5 – 1 mM range in cells, which indicates a partially functional energy metabolism. As NAD^+^ and ATP levels were not rescued to the same extent, axotomy-induced disturbance of energy metabolism seems to occur independently of NAD^+^ metabolism. Possibly, more than one interaction is required to fully rescue neurites.

## Conclusions and outlook

In this study, we demonstrated key processes controlling caspase-independent programmed neurite degeneration (PND) in human CNS neurons. We found that NAD^+^ metabolism, and in particular the NADase SARM1, played a key role not only in an axotomy model, but also e.g. in death triggered by the rodenticide Vacor. These finding suggest pharmacological or genetic inhibition of SARM1, and stabilization of NAD^+^ levels by metabolite supplementation or WLD(s) expression, as potential interventions against PND.

In our proof-of concept study, we triggered PND by axotomy or direct SARM1 activation to avoid ambiguities of more chronic disease models. However, we consider it likely that the compartmentalized cell death described here contributed also to more complex pathologies. For instance, mitochondrial complex I inhibitors can induce highly specific neurite damage in human neurons [50, 56, 64, 65], well in line with reports on mitochondrial dysfunction leading to SARM1 dependent neurite degeneration [37, 66]. In general, NAD^+^ is known to decline in the aging brain in mice [67] and in humans [68]. Disturbances in NAD^+^ metabolism are thought to be key drivers in brain aging and in neurodegenerative diseases [21]. For instance, NMNAT2 [69], and overall NAD^+^ levels [70, 71] are reduced in AD brains. NAD^+^ supplementation was found to be protective against several models of neurodegenerative diseases, including AD, PD, and ALS [21, 71, 72].

Another NAD^+^-consuming enzyme, PARP1, has long been thought to be involved in PD [73] and other neurodegenerative diseases [74]. PARP1 activation leads to generation poly(adenosine 5′-diphosphate–ribose) (PAR) and causes parthanatos [75]. It is however unclear if PAR acts as a gain-of-function death signal [76], or if a loss-of-function (NAD^+^ depletion by PARP1) causes cell death [77]. Similarly, it is still an open issue, whether SARM1 activation leads to neurite degeneration by detrimental NAD^+^ depletion, or by a gain-of-function, such as increased Ca^2+^-mobilization by the SARM1 product cADPR [78, 79].

SARM1 could be imagined as integrating sensor of metabolic disturbance in the axon - reduced NAD^+^ levels and mitochondrial dysfunction might increase the susceptibility to SARM1 activation, and therefore facilitate axonal degeneration in the CNS. To better understand the underlying regulation processes, further studies may need to provide real-time, single cell information on the metabolic state of neurites, or even on specific sub-structures. While genetically encoded sensors of ATP are well established [80], tools to detect NAD^+^ levels still need to be optimized [81]. Because of the complexity of SARM1 activation [82, 83], it is important to define exact activation thresholds for SARM1 by detailed metabolite measurements under different degenerative conditions. Here, we provide a relevant and accessible model to investigate these questions.

## Supplementary information

Bruell2024 supplementary material

Data Set 1

## Data Availability

All data are available from the corresponding author upon request.

## References

[1] Vickers JC, King AE, Woodhouse A, Kirkcaldie MT, Staal JA, McCormack GH, et al. Axonopathy and cytoskeletal disruption in degenerative diseases of the central nervous system. Brain Res Bull. 2009;80:217–23. 19683034 10.1016/j.brainresbull.2009.08.004

[2] Wang JT, Medress ZA, Barres BA. Axon degeneration: molecular mechanisms of a self-destruction pathway. J Cell Biol. 2012;196:7–18. 22232700 10.1083/jcb.201108111 PMC3255986

[3] Adalbert R, Coleman MP. Review: Axon pathology in age-related neurodegenerative disorders. Neuropathol Appl Neurobiol. 2013;39:90–108. 23046254 10.1111/j.1365-2990.2012.01308.x

[4] Burke RE, O’Malley K. Axon degeneration in Parkinson’s disease. Exp Neurol. 2013;246:72–83. 10.1016/j.expneurol.2012.01.011 PMC3340476 22285449

[5] Kanaan NM, Pigino GF, Brady ST, Lazarov O, Binder LI, Morfini GA. Axonal degeneration in Alzheimer’s disease: when signaling abnormalities meet the axonal transport system. Exp Neurol. 2013;246:44–53. 22721767 10.1016/j.expneurol.2012.06.003 PMC3465504

[6] Brady ST, Morfini GA. Regulation of motor proteins, axonal transport deficits and adult-onset neurodegenerative diseases. Neurobiol Dis. 2017;105:273–82. 28411118 10.1016/j.nbd.2017.04.010 PMC5522763

[7] Gcwensa NZ, Russell DL, Cowell RM, Volpicelli-Daley LA. Molecular mechanisms underlying synaptic and axon degeneration in parkinson’s disease. Front Cell Neurosci. 2021;15:626128. 33737866 10.3389/fncel.2021.626128 PMC7960781

[8] Athauda D, Foltynie T. The ongoing pursuit of neuroprotective therapies in Parkinson disease. Nat Rev Neurol. 2015;11:25–40. 25447485 10.1038/nrneurol.2014.226

[9] Dhani S, Zhao Y, Zhivotovsky B. A long way to go: caspase inhibitors in clinical use. Cell Death Dis. 2021;12:949. 34654807 10.1038/s41419-021-04240-3 PMC8519909

[10] Sagot Y, Dubois-Dauphin M, Tan SA, de Bilbao F, Aebischer P, Martinou JC, et al. Bcl-2 overexpression prevents motoneuron cell body loss but not axonal degeneration in a mouse model of a neurodegenerative disease. J Neurosci. 1995;15:7727–33. 7472523 10.1523/JNEUROSCI.15-11-07727.1995 PMC6578059

[11] Waldmeier P, Bozyczko-Coyne D, Williams M, Vaught JL. Recent clinical failures in Parkinson’s disease with apoptosis inhibitors underline the need for a paradigm shift in drug discovery for neurodegenerative diseases. Biochem Pharm. 2006;72:1197–206. 16901468 10.1016/j.bcp.2006.06.031

[12] Coleman MP, Hoke A. Programmed axon degeneration: from mouse to mechanism to medicine. Nat Rev Neurosci. 2020;21:183–96. 32152523 10.1038/s41583-020-0269-3 PMC8926152

[13] Waller A. Experiments on the section of the glosso-pharyngeal and hypoglossal nerves of the frog, and observations of the alterations produced thereby in the structure of their primitive fibres. Edinb Med Surg J. 1851;76:369–76. 30332247 PMC5929074

[14] Mack TG, Reiner M, Beirowski B, Mi W, Emanuelli M, Wagner D, et al. Wallerian degeneration of injured axons and synapses is delayed by a Ube4b/Nmnat chimeric gene. Nat Neurosci. 2001;4:1199–206. 11770485 10.1038/nn770

[15] Wang M, Wu Y, Culver DG, Glass JD. The gene for slow Wallerian degeneration (Wld(s)) is also protective against vincristine neuropathy. Neurobiol Dis. 2001;8:155–61. 11162249 10.1006/nbdi.2000.0334

[16] Wang MS, Fang G, Culver DG, Davis AA, Rich MM, Glass JD. The WldS protein protects against axonal degeneration: a model of gene therapy for peripheral neuropathy. Ann Neurol. 2001;50:773–9. 11761475 10.1002/ana.10039

[17] Lunn ER, Perry VH, Brown MC, Rosen H, Gordon S. Absence of Wallerian degeneration does not hinder regeneration in peripheral nerve. Eur J Neurosci. 1989;1:27–33. 12106171 10.1111/j.1460-9568.1989.tb00771.x

[18] Gilley J, Coleman MP. Endogenous Nmnat2 is an essential survival factor for maintenance of healthy axons. PLoS Biol. 2010;8:e1000300. 20126265 10.1371/journal.pbio.1000300 PMC2811159

[19] Coleman MP, Freeman MR. Wallerian degeneration, wld(s), and nmnat. Annu Rev Neurosci. 2010;33:245–67. 20345246 10.1146/annurev-neuro-060909-153248 PMC5223592

[20] Figley MD, DiAntonio A. The SARM1 axon degeneration pathway: control of the NAD(+) metabolome regulates axon survival in health and disease. Curr Opin Neurobiol. 2020;63:59–66. 32311648 10.1016/j.conb.2020.02.012 PMC7483800

[21] Lautrup S, Sinclair DA, Mattson MP, Fang EF. NAD(+) in brain aging and neurodegenerative disorders. Cell Metab. 2019;30:630–55. 31577933 10.1016/j.cmet.2019.09.001 PMC6787556

[22] Essuman K, Summers DW, Sasaki Y, Mao X, DiAntonio A, Milbrandt J. The SARM1 toll/interleukin-1 receptor domain possesses intrinsic NAD(+) cleavage activity that promotes pathological axonal degeneration. Neuron. 2017;93:1334–43.e5. 28334607 10.1016/j.neuron.2017.02.022 PMC6284238

[23] Gerdts J, Brace EJ, Sasaki Y, DiAntonio A, Milbrandt J. SARM1 activation triggers axon degeneration locally via NAD(+) destruction. Science. 2015;348:453–7. 25908823 10.1126/science.1258366 PMC4513950

[24] Loreto A, Di Stefano M, Gering M, Conforti L. Wallerian degeneration is executed by an NMN-SARM1-dependent late Ca(2+) influx but only modestly influenced by mitochondria. Cell Rep. 2015;13:2539–52. 26686637 10.1016/j.celrep.2015.11.032

[25] Osterloh JM, Yang J, Rooney TM, Fox AN, Adalbert R, Powell EH, et al. dSarm/Sarm1 is required for activation of an injury-induced axon death pathway. Science. 2012;337:481–4. 22678360 10.1126/science.1223899 PMC5225956

[26] Di Stefano M, Nascimento-Ferreira I, Orsomando G, Mori V, Gilley J, Brown R, et al. A rise in NAD precursor nicotinamide mononucleotide (NMN) after injury promotes axon degeneration. Cell Death Differ. 2015;22:731–42. 25323584 10.1038/cdd.2014.164 PMC4392071

[27] Zhao ZY, Xie XJ, Li WH, Liu J, Chen Z, Zhang B, et al. A cell-permeant mimetic of NMN activates SARM1 to produce cyclic ADP-ribose and induce non-apoptotic cell death. iScience. 2019;15:452–66. 31128467 10.1016/j.isci.2019.05.001 PMC6531917

[28] Jiang Y, Liu T, Lee CH, Chang Q, Yang J, Zhang Z. The NAD(+)-mediated self-inhibition mechanism of pro-neurodegenerative SARM1. Nature. 2020;588:658–63. 33053563 10.1038/s41586-020-2862-z

[29] Sporny M, Guez-Haddad J, Khazma T, Yaron A, Dessau M, Shkolnisky Y, et al. Structural basis for SARM1 inhibition and activation under energetic stress. Elife. 2020;9:e62021. 10.7554/eLife.62021 PMC7688312 33185189

[30] Figley MD, Gu W, Nanson JD, Shi Y, Sasaki Y, Cunnea K, et al. SARM1 is a metabolic sensor activated by an increased NMN/NAD(+) ratio to trigger axon degeneration. Neuron. 2021;109:1118–36.e11. 33657413 10.1016/j.neuron.2021.02.009 PMC8174188

[31] Ko KW, Devault L, Sasaki Y, Milbrandt J, DiAntonio A. Live imaging reveals the cellular events downstream of SARM1 activation. Elife. 2021;10:e71148. 10.7554/eLife.71148 PMC8612704 34779400

[32] Buonvicino D, Mazzola F, Zamporlini F, Resta F, Ranieri G, Camaioni E, et al. Identification of the nicotinamide salvage pathway as a new toxification route for antimetabolites. Cell Chem Biol. 2018;25:471–82.e7. 29478906 10.1016/j.chembiol.2018.01.012

[33] Loreto A, Angeletti C, Gu W, Osborne A, Nieuwenhuis B, Gilley J, et al. Neurotoxin-mediated potent activation of the axon degeneration regulator SARM1. Elife. 2021;10:e72823. 10.7554/eLife.72823 PMC8758145 34870595

[34] Wu T, Zhu J, Strickland A, Ko KW, Sasaki Y, Dingwall CB, et al. Neurotoxins subvert the allosteric activation mechanism of SARM1 to induce neuronal loss. Cell Rep. 2021;37:109872. 34686345 10.1016/j.celrep.2021.109872 PMC8638332

[35] Kitay BM, McCormack R, Wang Y, Tsoulfas P, Zhai RG. Mislocalization of neuronal mitochondria reveals regulation of Wallerian degeneration and NMNAT/WLD(S)-mediated axon protection independent of axonal mitochondria. Hum Mol Genet. 2013;22:1601–14. 23314018 10.1093/hmg/ddt009 PMC3657477

[36] Chen YH, Sasaki Y, DiAntonio A, Milbrandt J. SARM1 is required in human derived sensory neurons for injury-induced and neurotoxic axon degeneration. Exp Neurol. 2021;339:113636. 33548217 10.1016/j.expneurol.2021.113636 PMC8171232

[37] Hughes RO, Bosanac T, Mao X, Engber TM, DiAntonio A, Milbrandt J, et al. Small molecule SARM1 inhibitors recapitulate the SARM1(−/−) phenotype and allow recovery of a metastable pool of axons fated to degenerate. Cell Rep. 2021;34:108588. 33406435 10.1016/j.celrep.2020.108588 PMC8179325

[38] Hinz FI, Villegas CLM, Roberts JT, Yao H, Gaddam S, Delwig A, et al. Context-specific stress causes compartmentalized SARM1 activation and local degeneration in cortical neurons. J Neurosci. 2024;44:e2424232024. 10.1523/JNEUROSCI.2424-23.2024 PMC11170950 38692735

[39] Loreto A, Cramb KML, McDermott LA, Antoniou C, Cirilli I, Caiazza MC, et al. SARM1 activation induces reversible mitochondrial dysfunction and can be prevented in human neurons by antisense oligonucleotides. bioRxiv. 2024. 10.1101/2024.04.02.587827. 10.1016/j.nbd.2025.106986 PMC7617922 40473123

[40] Loreto A, Merlini E, Coleman MP. Programmed axon death: a promising target for treating retinal and optic nerve disorders. Eye (Lond). 2024;38:1802–9. 10.1038/s41433-024-03025-0 PMC11226669 38538779

[41] Henninger N, Bouley J, Sikoglu EM, An J, Moore CM, King JA, et al. Attenuated traumatic axonal injury and improved functional outcome after traumatic brain injury in mice lacking Sarm1. Brain. 2016;139:1094–105. 26912636 10.1093/brain/aww001 PMC5006226

[42] Ziogas NK, Koliatsos VE. Primary traumatic axonopathy in mice subjected to impact acceleration: a reappraisal of pathology and mechanisms with high-resolution anatomical methods. J Neurosci. 2018;38:4031–47. 29567804 10.1523/JNEUROSCI.2343-17.2018 PMC6705930

[43] Hasbani DM. O’Malley KL. Wld(S) mice are protected against the Parkinsonian mim etic MPTP. Exp Neurol. 2006;202:93–9. 10.1016/j.expneurol.2006.05.017 16806180

[44] Sajadi A, Schneider BL, Aebischer P. Wlds-mediated protection of dopaminergic fibers in an animal model of Parkinson disease. Curr Biol. 2004;14:326–30. 14972684 10.1016/j.cub.2004.01.053

[45] Miyamoto T, Kim C, Chow J, Dugas JC, DeGroot J, Bagdasarian AL, et al. SARM1 is responsible for calpain-dependent dendrite degeneration in mouse hippocampal neurons. J Biol Chem. 2024;300:105630. 38199568 10.1016/j.jbc.2024.105630 PMC10862016

[46] Conforti L, Gilley J, Coleman MP. Wallerian degeneration: an emerging axon death pathway linking injury and disease. Nat Rev Neurosci. 2014;15:394–409. 24840802 10.1038/nrn3680

[47] Krauss R, Bosanac T, Devraj R, Engber T, Hughes RO. Axons matter: the promise of treating neurodegenerative disorders by targeting sarm1-mediated axonal degeneration. Trends Pharm Sci. 2020;41:281–93. 32107050 10.1016/j.tips.2020.01.006

[48] Brull M, Geese N, Celardo I, Laumann M, Leist M. Preparation of Viable Human Neurites for Neurobiological and Neurodegeneration Studies. Cells. 2024;13:242. 10.3390/cells13030242 PMC10854604 38334634

[49] Delp J, Gutbier S, Klima S, Hoelting L, Pinto-Gil K, Hsieh JH, et al. A high-throughput approach to identify specific neurotoxicants/ developmental toxicants in human neuronal cell function assays. ALTEX. 2018;35:235–53. 29423527 10.14573/altex.1712182 PMC10266261

[50] Krug AK, Balmer NV, Matt F, Schonenberger F, Merhof D, Leist M. Evaluation of a human neurite growth assay as specific screen for developmental neurotoxicants. Arch Toxicol. 2013;87:2215–31. 23670202 10.1007/s00204-013-1072-y

[51] Lotharius J, Falsig J, van Beek J, Payne S, Dringen R, Brundin P, et al. Progressive degeneration of human mesencephalic neuron-derived cells triggered by dopamine-dependent oxidative stress is dependent on the mixed-lineage kinase pathway. J Neurosci. 2005;25:6329–42. 16000623 10.1523/JNEUROSCI.1746-05.2005 PMC6725277

[52] Suciu I, Delp J, Gutbier S, Suess J, Henschke L, Celardo I, et al. Definition of the neurotoxicity-associated metabolic signature triggered by berberine and other respiratory chain inhibitors. Antioxidants (Basel). 2023;13:49. 10.3390/antiox13010049 PMC10812665 38247474

[53] Brull M, Spreng AS, Gutbier S, Loser D, Krebs A, Reich M, et al. Incorporation of stem cell-derived astrocytes into neuronal organoids to allow neuro-glial interactions in toxicological studies. ALTEX. 2020;37:409–28. 32150624 10.14573/altex.1911111

[54] Scholz D, Poltl D, Genewsky A, Weng M, Waldmann T, Schildknecht S, et al. Rapid, complete and large-scale generation of post-mitotic neurons from the human LUHMES cell line. J Neurochem. 2011;119:957–71. 21434924 10.1111/j.1471-4159.2011.07255.x

[55] Gutbier S, May P, Berthelot S, Krishna A, Trefzer T, Behbehani M, et al. Major changes of cell function and toxicant sensitivity in cultured cells undergoing mild, quasi-natural genetic drift. Arch Toxicol. 2018;92:3487–503. 30298209 10.1007/s00204-018-2326-5 PMC6290691

[56] Stiegler NV, Krug AK, Matt F, Leist M. Assessment of chemical-induced impairment of human neurite outgrowth by multiparametric live cell imaging in high-density cultures. Toxicol Sci. 2011;121:73–87. 21342877 10.1093/toxsci/kfr034

[57] Uckert AK, Rutschlin S, Gutbier S, Worz NC, Miah MR, Martins AC, et al. Identification of the bacterial metabolite aerugine as potential trigger of human dopaminergic neurodegeneration. Environ Int. 2023;180:108229. 37797477 10.1016/j.envint.2023.108229 PMC10666548

[58] Schildknecht S, Karreman C, Poltl D, Efremova L, Kullmann C, Gutbier S, et al. Generation of genetically-modified human differentiated cells for toxicological tests and the study of neurodegenerative diseases. ALTEX. 2013;30:427–44. 24173167 10.14573/altex.2013.4.427

[59] Geisler S, Huang SX, Strickland A, Doan RA, Summers DW, Mao X, et al. Gene therapy targeting SARM1 blocks pathological axon degeneration in mice. J Exp Med. 2019;216:294–303. 30642945 10.1084/jem.20181040 PMC6363435

[60] Braselmann S, Graninger P, Busslinger M. A selective transcriptional induction system for mammalian cells based on Gal4-estrogen receptor fusion proteins. Proc Natl Acad Sci USA. 1993;90:1657–61. 8446579 10.1073/pnas.90.5.1657 PMC45938

[61] Matentzoglu K, Scheffner M. Ubiquitin-fusion protein system: a powerful tool for ectopic protein expression in mammalian cells. Biotechniques. 2009;46:21–2. 19301619 10.2144/000113023

[62] Karreman C. AiO, combining DNA/protein programs and oligo-management. Bioinformatics. 2002;18:884–5. 12075025 10.1093/bioinformatics/18.6.884

[63] Shi Y, Kerry PS, Nanson JD, Bosanac T, Sasaki Y, Krauss R, et al. Structural basis of SARM1 activation, substrate recognition, and inhibition by small molecules. Mol Cell. 2022;82:1643–59.e10. 35334231 10.1016/j.molcel.2022.03.007 PMC9188649

[64] Delp J, Cediel-Ulloa A, Suciu I, Kranaster P, van Vugt-Lussenburg BM, Munic Kos V, et al. Neurotoxicity and underlying cellular changes of 21 mitochondrial respiratory chain inhibitors. Arch Toxicol. 2021;95:591–615. 33512557 10.1007/s00204-020-02970-5 PMC7870626

[65] Delp J, Funke M, Rudolf F, Cediel A, Bennekou SH, van der Stel W, et al. Development of a neurotoxicity assay that is tuned to detect mitochondrial toxicants. Arch Toxicol. 2019;93:1585–608. 31190196 10.1007/s00204-019-02473-y

[66] Summers DW, DiAntonio A, Milbrandt J. Mitochondrial dysfunction induces Sarm1-dependent cell death in sensory neurons. J Neurosci. 2014;34:9338–50. 25009267 10.1523/JNEUROSCI.0877-14.2014 PMC4087211

[67] Stein LR, Imai S. Specific ablation of Nampt in adult neural stem cells recapitulates their functional defects during aging. EMBO J. 2014;33:1321–40. 24811750 10.1002/embj.201386917 PMC4194122

[68] Zhu XH, Lu M, Lee BY, Ugurbil K, Chen W. In vivo NAD assay reveals the intracellular NAD contents and redox state in healthy human brain and their age dependences. Proc Natl Acad Sci USA. 2015;112:2876–81. 25730862 10.1073/pnas.1417921112 PMC4352772

[69] Ali YO, Allen HM, Yu L, Li-Kroeger D, Bakhshizadehmahmoudi D, Hatcher A, et al. NMNAT2:HSP90 complex mediates proteostasis in proteinopathies. PLoS Biol. 2016;14:e1002472. 27254664 10.1371/journal.pbio.1002472 PMC4890852

[70] Dong Y, Brewer GJ. Global metabolic shifts in age and alzheimer’s disease mouse brains pivot at NAD+/NADH redox sites. J Alzheimers Dis. 2019;71:119–40. 31356210 10.3233/JAD-190408 PMC6839468

[71] Hou Y, Lautrup S, Cordonnier S, Wang Y, Croteau DL, Zavala E, et al. NAD(+) supplementation normalizes key Alzheimer’s features and DNA damage responses in a new AD mouse model with introduced DNA repair deficiency. Proc Natl Acad Sci USA. 2018;115:E1876–E85. 29432159 10.1073/pnas.1718819115 PMC5828618

[72] Lehmann S, Costa AC, Celardo I, Loh SH, Martins LM. Parp mutations protect against mitochondrial dysfunction and neurodegeneration in a PARKIN model of Parkinson’s disease. Cell Death Dis. 2016;7:e2166. 27031963 10.1038/cddis.2016.72 PMC4823968

[73] Kam TI, Mao X, Park H, Chou SC, Karuppagounder SS, Umanah GE, et al. Poly(ADP-ribose) drives pathologic alpha-synuclein neurodegeneration in Parkinson’s disease. Science. 2018;362:eaat8407. 10.1126/science.aat8407 PMC6431793 30385548

[74] Yang L, Guttman L, Dawson VL, Dawson TM Parthanatos: Mechanisms, modulation, and therapeutic prospects in neurodegenerative disease and stroke. Biochem Pharmacol. 2024:228:116174. 10.1016/j.bcp.2024.116174 PMC11410548 38552851

[75] Park H, Kam TI, Dawson TM, Dawson VL. Poly (ADP-ribose) (PAR)-dependent cell death in neurodegenerative diseases. Int Rev Cell Mol Biol. 2020;353:1–29. 32381174 10.1016/bs.ircmb.2019.12.009

[76] Andrabi SA, Kim NS, Yu SW, Wang H, Koh DW, Sasaki M, et al. Poly(ADP-ribose) (PAR) polymer is a death signal. Proc Natl Acad Sci USA. 2006;103:18308–13. 17116882 10.1073/pnas.0606526103 PMC1838747

[77] Alano CC, Garnier P, Ying W, Higashi Y, Kauppinen TM, Swanson RA. NAD+ depletion is necessary and sufficient for poly(ADP-ribose) polymerase-1-mediated neuronal death. J Neurosci. 2010;30:2967–78. 20181594 10.1523/JNEUROSCI.5552-09.2010 PMC2864043

[78] Sasaki Y, Engber TM, Hughes RO, Figley MD, Wu T, Bosanac T, et al. cADPR is a gene dosage-sensitive biomarker of SARM1 activity in healthy, compromised, and degenerating axons. Exp Neurol. 2020;329:113252. 32087251 10.1016/j.expneurol.2020.113252 PMC7302925

[79] Li Y, Pazyra-Murphy MF, Avizonis D, de Sa Tavares Russo M, Tang S, Chen CY, et al. Sarm1 activation produces cADPR to increase intra-axonal Ca++ and promote axon degeneration in PIPN. J Cell Biol. 2022;221:e202106080. 10.1083/jcb.202106080 PMC8704956 34935867

[80] White D, 3rd, Yang Q. Genetically encoded ATP biosensors for direct monitoring of cellular ATP dynamics. Cells. 2022;11:1920. 10.3390/cells11121920 PMC9221525 35741049

[81] Cohen MS, Stewart ML, Goodman RH, Cambronne XA. Methods for using a genetically encoded fluorescent biosensor to monitor nuclear NAD+. Methods Mol Biol. 2018;1813:391–414. 30097882 10.1007/978-1-4939-8588-3_26 PMC6378224

[82] Liu HW, Smith CB, Schmidt MS, Cambronne XA, Cohen MS, Migaud ME, et al. Pharmacological bypass of NAD(+) salvage pathway protects neurons from chemotherapy-induced degeneration. Proc Natl Acad Sci USA. 2018;115:10654–9. 30257945 10.1073/pnas.1809392115 PMC6196523

[83] Sasaki Y, Zhu J, Shi Y, Gu W, Kobe B, Ve T, et al. Nicotinic acid mononucleotide is an allosteric SARM1 inhibitor promoting axonal protection. Exp Neurol. 2021;345:113842. 34403688 10.1016/j.expneurol.2021.113842 PMC8571713

