# Supplementary material for: Programmed neurite degeneration in human central nervous system neurons driven by changes in NAD+ metabolism
Source: Cell Death Dis. 2025 Jan 17;16(1):24. doi: 10.1038/s41419-024-07326-w (PMC11742042; doi:10.1038/s41419-024-07326-w)
Supplement: Supplementary file 1 — Bruell2024 supplementary material [file 41419_2024_7326_MOESM1_ESM.docx]

*Supplementary information for*

Programmed neurite degeneration in human central nervous system neurons driven by changes in NAD^+^ metabolism

Markus Brüll, Selina Multrus, Michael Schäfer, Ivana Celardo, Christiaan Karreman and Marcel Leist

| Table of Contents | | |
| --- | --- | --- |
| Figure S1 | Schematic representation of the experimental setup and metabolic control of SARM1 activity | 2 |
| Figure S2 | Energy metabolism in isolated neurites | 3 |
| Figure S3 | Quantification of western blots | 4 |
| Figure S4 | Knockdown of SARM1 in monolayer LUHMES cells | 5 |
| Figure S5 | Knockdown of SARM1 in LUHMES neurites | 6 |
| Figure S6 | dnSARM1-expressing LUHMES cells | 7 |
| Figure S7 | ATP and NAD levels in WLD(s)-expressing neurites | 8 |
| Table S1 | Chemicals and media supplements used in this study. | 9 |
| Table S2 | List of oligonucleotides used for the creation of dnSARM1 | 10 |
| Table S3 | List of antibodies used in this study. | 10 |

**Supplementary figure 1: Schematic representation of the experimental setup and metabolic control of SARM1 activity. A:** To generate isolated neurite cultures, LUHMES cells were differentiated for two days in T75 flasks. On DoD2, cells were seeded into ultra-low attachment round bottom plates for spheroid generation. Spheroids were seeded on Matrigel-coated flat-bottom plates on DoD9 to grow out neurites. Axotomy was typically induced on DoD14 with or without intervention. Neurites were analyzed at different time points after axotomy. **B:** Schematic representation of metabolic control of SARM1. In intact neurites, NAD^+^ is synthesized from vitamin B3/nicotinamide (Nam) via the intermediate product nicotinamide mononucleotide (NMN). In the presence of NMNAT2, NMN levels are low and the high level of NAD^+^ is high. This prevents SARM1 activation. After axotomy, NMNAT2 is rapidly lost. This leads to an accumulation of NMN and a decrease of NAD^+^. This altered balance promotes SARM1 activity. Box sizes suggest levels of the metabolites; grey arrows indicate metabolic conversion; green/red arrows indicate activation/inhibition; enzymes in green ovals are considered active, enzymes in red ovals are deemed to be inactive. **C:** An increased Nam concentration inhibits SARM1 by feedback inhibition. Thus, Nam supplementation is supposed to reduce SARM1 activity. Further increasing Nam by NAMPT inhibition by FK866 is not likely to affect the anyway dampened SARM1 activity. To test this, plated spheroids were treated with different Nam concentrations immediately before axotomy. Neurites were stained with calcein-AM 18 h later. Neurite integrity and fragmentation were quantified. Cells were treated with Nam alone or in the presence of 500 nM FK866 (dotted lines). Note: Fig 1 shows that FK866 is pharmacologically active at 500 nM concentration. Note also: The simplified metabolic scheme in Fig. 1I was designed assuming that there was no NMNAT activity (situation after axotomy; see Fig. 1D).


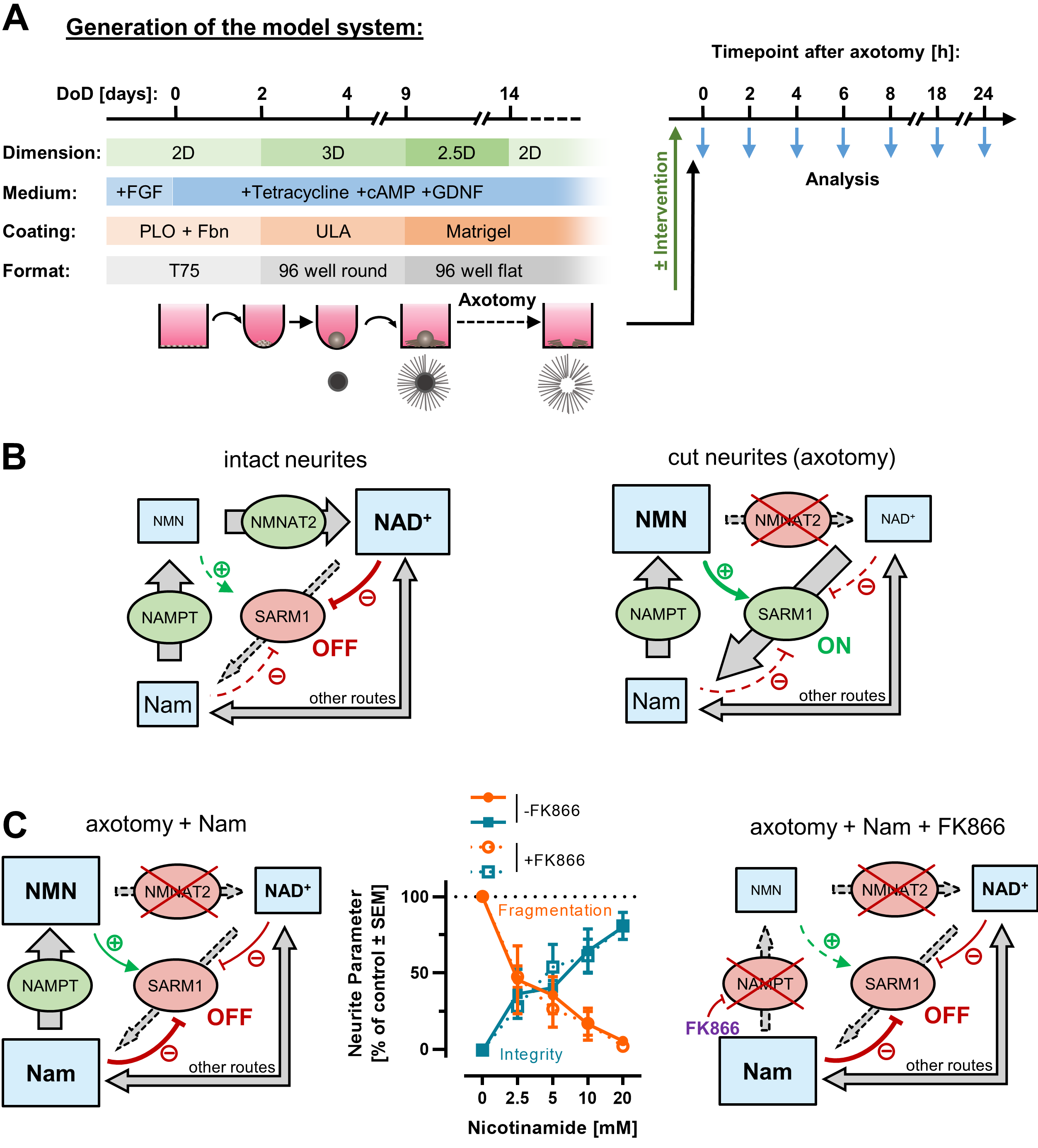

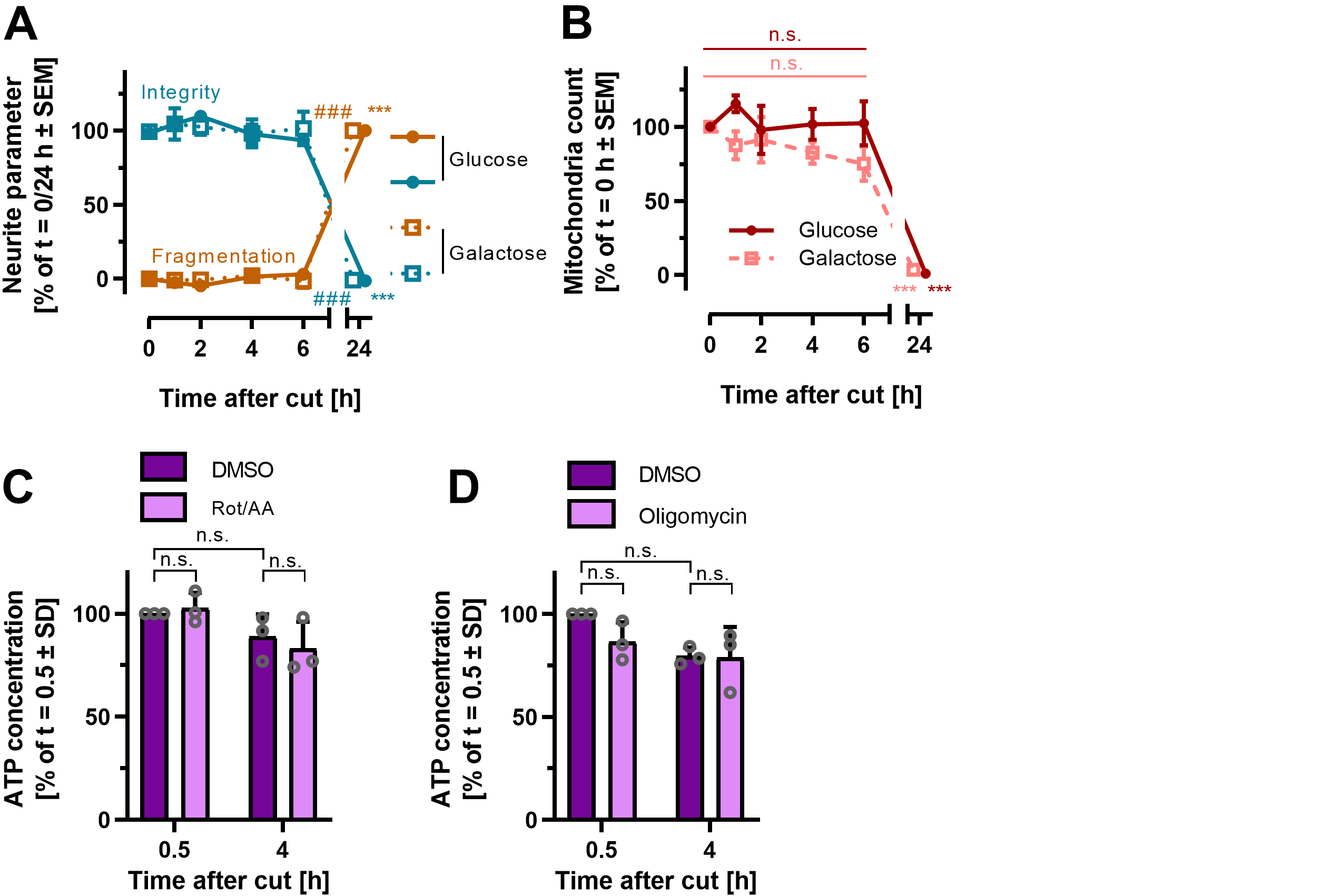


**Supplementary figure 2: Energy metabolism in isolated neurites. A/B:** Cells were either cultured in standard medium (glucose containing), or in medium containing galactose instead of glucose (not allowing for glycolytic ATP production). Neurites were cut at different time points and stained with calcein-AM and TMRE and imaged by epifluorescence microscopy. Neurite integrity, fragmentation (A) and the number of TMRE^+^ mitochondria (mitochondria count; normalized to the neurite area) (B) was quantified in isolated neurites at different time points after axotomy. ***/### = p < 0.001 analyzed by ANOVA with Dunnet’s *post hoc* test. **C/D:** The ATP content of neurites was measured 30 min or 4 h after axotomy. The experiment was performed in presence or absence of mitochondrial inhibitors (C: rotenone/antimycin A, (Rot/AA); D: oligomycin), added for 30 min before sampling. Data are means from biological replicates. Cf. Fig 2 for same experiment, with glycolysis inhibited. Significance was evaluated by two-way ANOVA followed by Tukey’s *post hoc* test. n.s. = not significant.


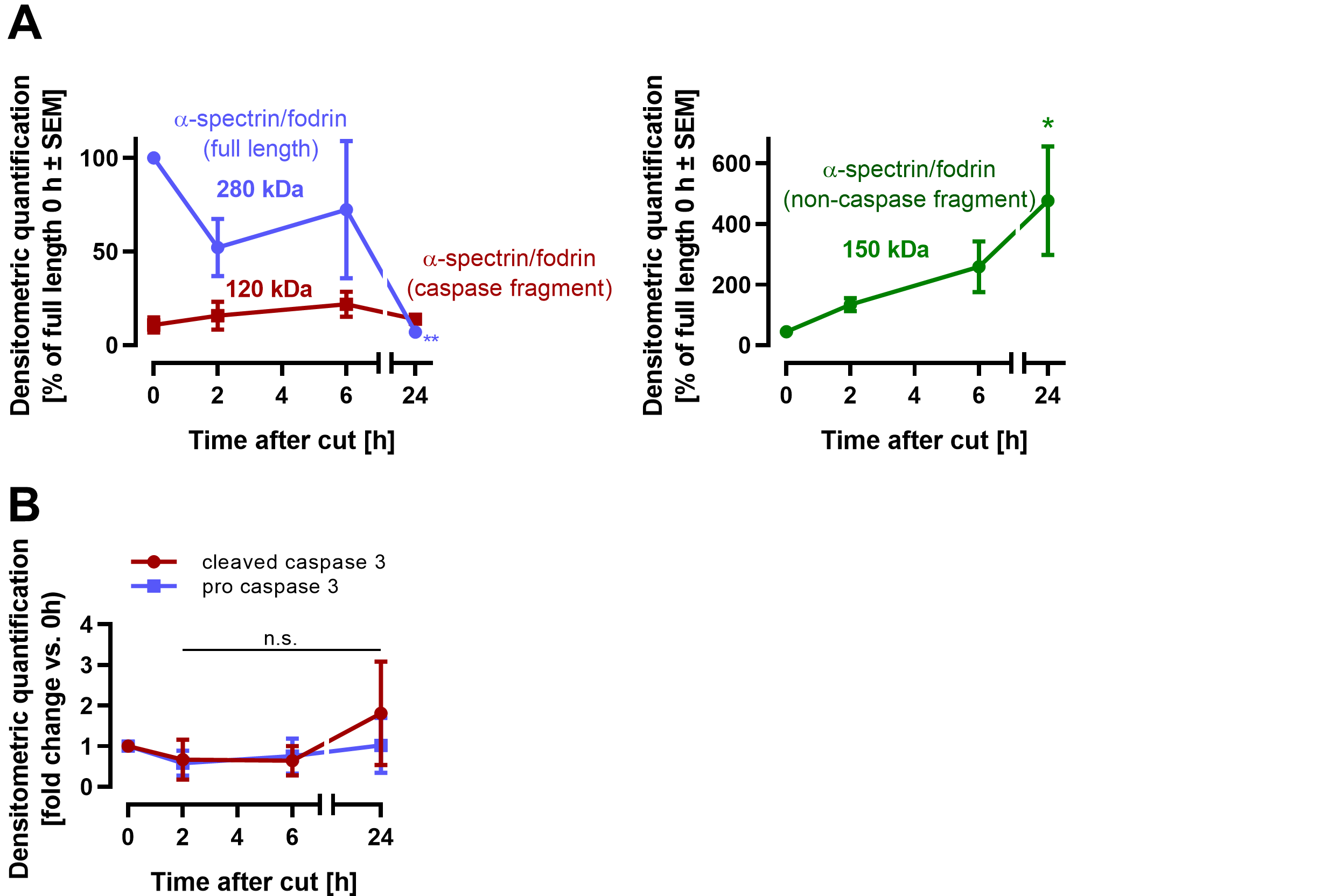


**Supplementary figure 3: Quantification of western blots. A:** Densitometric quantification of αII-spectrin western blot bands shown in Fig. 3D. Bands were normalized to the intensity of respective loading controls. Full length αII-spectrin was set to 100%. Data are from three independent experiments. **B:** Densitometric quantification of caspase 3 western blot bands shown in Fig. 3E. Bands were normalized to the intensity of respective loading controls. Data are from three independent experiments.


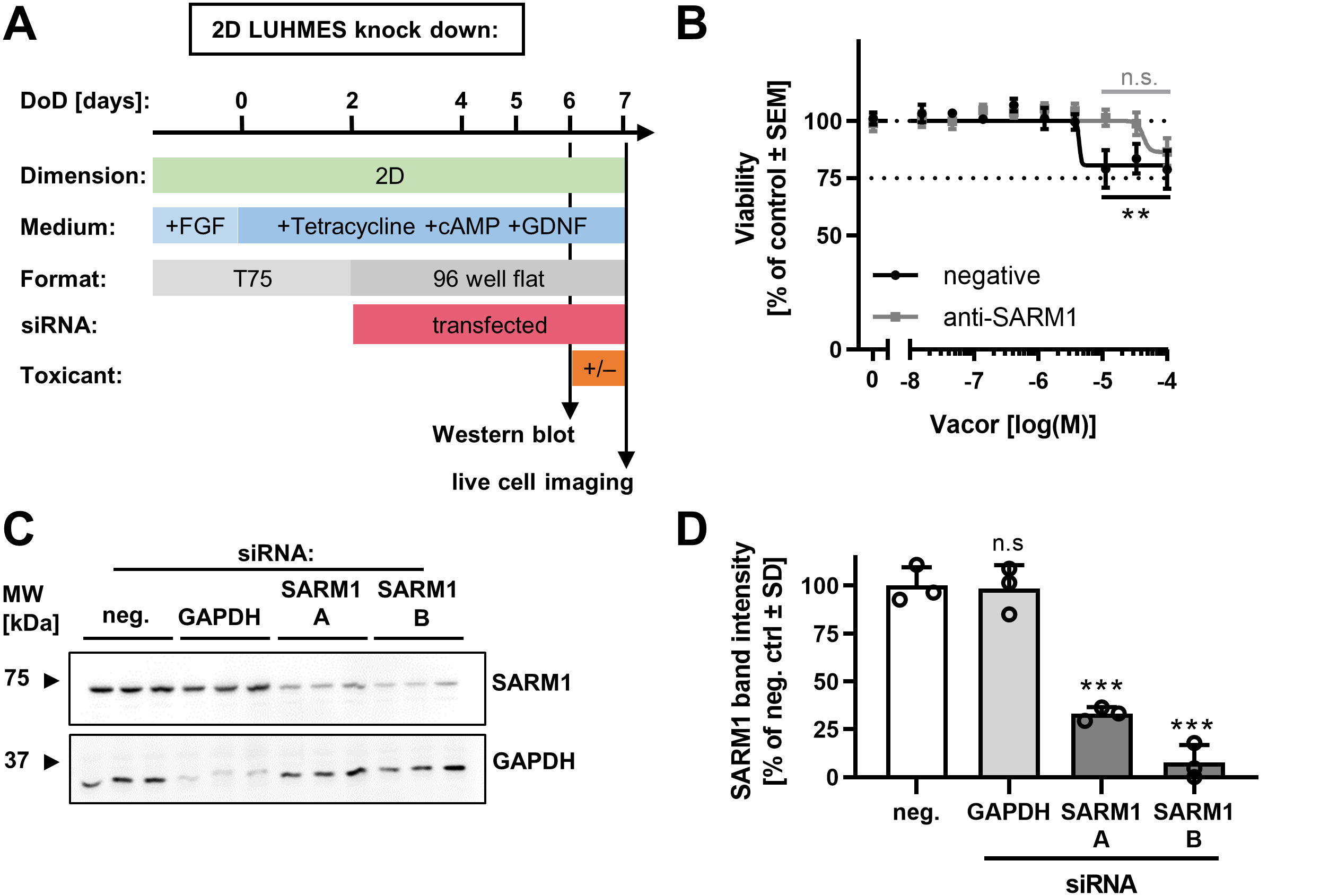


**Supplementary figure 4: Knockdown of SARM1 in monolayer LUHMES cells. A:** To obtain an efficient SARM1 knockdown in differentiated LUHMES cells, the following protocol was optimized: Cells were differentiated for two days in T75 flasks and detached. Before reseeding, these DoD2 cells were transfected in suspension with siRNAs. Cells were seeded into flat-bottom 96 well plates. Transfected cells were then left untreated until DoD6. Then they were lysed; SARM1 protein levels were analyzed by Western blotting in the cell lysates. To obtain a functional readout, transfected cells were treated on DoD6 with vacor and imaged 18 h later for viability and neurite integrity. **B:** Corresponding cell viability curves for Fig. 5C. Transfected cells were treated on DoD6 with different vacor concentrations. Cells were stained with calcein-AM and H-33342 and imaged by epifluorescence microscopy. Cell viability was determined by an image analysis algorithm counting Hoechst/Calcein double positive cells. Data was normalized to untreated controls. n.s. = not significant, ** = p < 0.01 by ANOVA with Dunnet’s *post hoc* test. **C:** LUHMES cells were transfected with negative (untargeted) siRNA, siRNA targeting GAPDH (positive control), or siRNA targeting different regions in the SARM1 transcript (SARM1 A (ThermoFisher Silencer Select; ID: s23032) and SARM1 B (ThermoFisher Silencer Select; ID: s23031)). Cells were transfected on DoD2 and sampled on DoD6. Each band represents an independent biological replicate. Because of the higher efficacy, the “SARM1 B” siRNA was used for all further experiments. **D:** Densitometric quantification of the western blot shown in C. Each point represents an independent biological replicate.


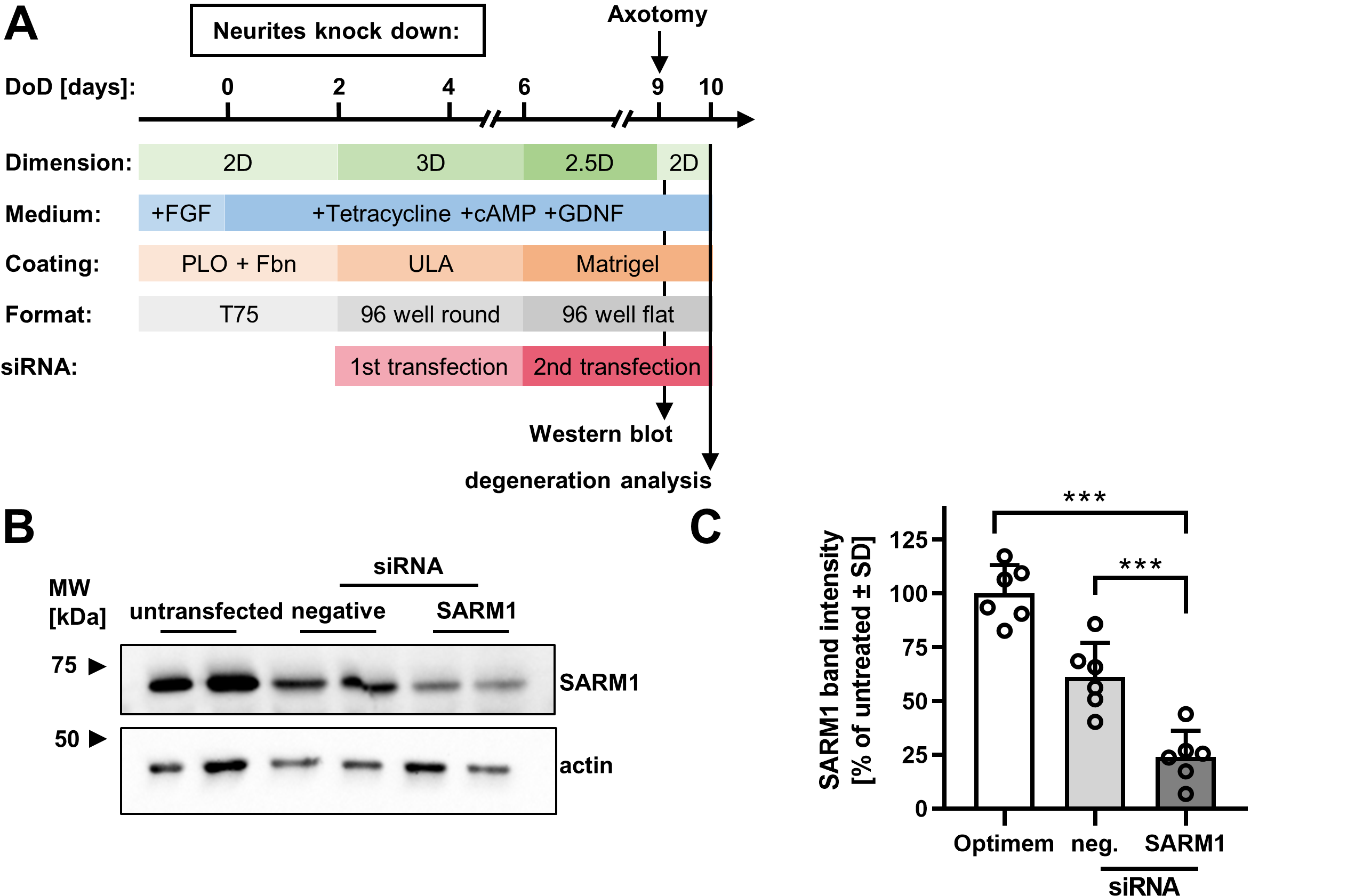


**Supplementary figure 5: Knockdown of SARM1 in LUHMES neurites. A:** Schematic describing the procedure used to obtain SARM1 knockdown in LUHMES neurites derived from spheroids. In brief, a shortened protocol was used to generate 2.5D cultures (and isolated neurites derived from these). Moreover, LUHMES cells were transfected twice. Cells were transfected in suspension on DoD2 and as spheroids on DoD6 during plating. Cells were transfected with either negative, untargeted siRNA, or anti-SARM1 siRNA (SARM1 B). On DoD9, neurites were isolated for Western blot generation, or isolated neurites were imaged 18 h after axotomy (Fig. 5D-F). **B:** Neurites were isolated on DoD9 and cell lysates were prepared. For each sample, 5 wells were pooled. Duplicate bands represent technical replicates (5 wells each). One of three blots of independent experiments is shown. **C:** Densitometric quantification of the SARM1 western blot described in B. SARM1 band intensity was normalized to corresponding actin bands. All data are normalized to the Optimem condition (ie. Transfection procedure without any siRNA or Lipofectamine added) Data from three biological replicates, with two technical replicates each, is shown. *** = p < 0.001 by ANOVA with Tukey’s *post hoc* test.


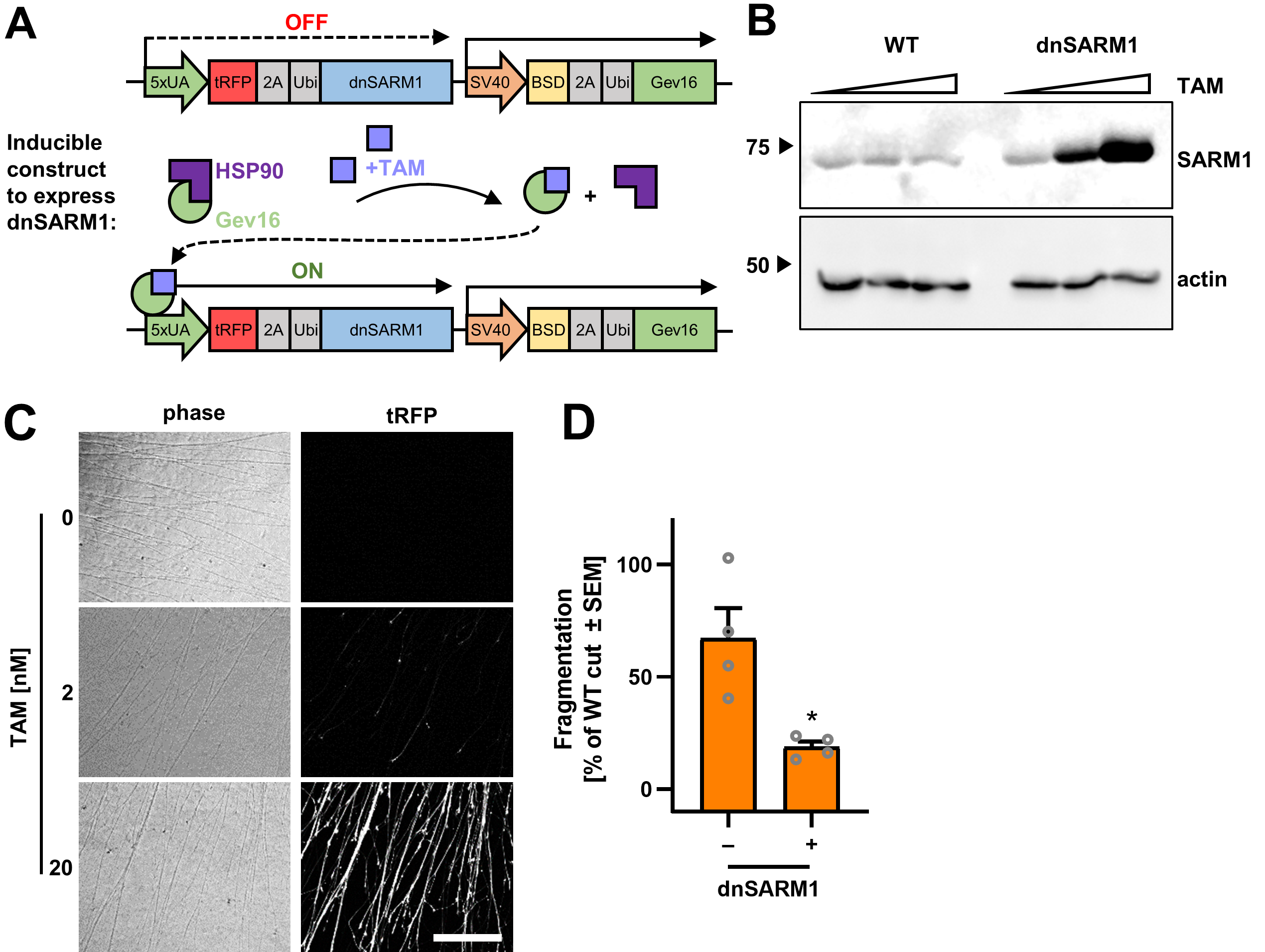


**Supplementary figure 6: dnSARM1-expressing LUHMES cells. A:** Schematic representation of the inducible construct integrated into LUHMES cells. A blasticidin S resistance gene and Gev16 are constitutively expressed under the control of a SV40 promotor. Gev16 is a fusion protein of the yeast transcription factor Gal4, the hormone binding domain of the human estrogen receptor, and the strong activating domain of the herpesvirus protein VP16 (Braselmann *et al.* 1993). The use of a 2A/Ubi (2A sequence of porcine teschovirus-1/ubiquitin) linker allowed the (post)translational separation of the BSD and Gev16 proteins. Without 4-OH-tamoxifene (TAM), Gev16 remains bound to HSP90 in the cytosol. Upon addition of TAM, Gev16 is released from HSP90 and can translocate into the nucleus. In the nucleus, it binds the upstream activating sequence (5xUA), which triggers the transcription of turbo red fluorescent protein (tRFP) and dominant negative SARM1 (dnSARM1) mRNA. The 2A/Ubi linker ensures separation of the two proteins by normal cellular ubiquitin hydrolases. **B:** Expression of dnSARM1 was induced in LUHMES spheroids on DoD9. As a control, WT spheroids were treated with TAM. Neurites were isolated on DoD15. Ten wells were pooled per condition. The triangle symbolizes the increasing TAM concentrations used: 0 nM, 2 nM, and 20 nM. An anti- SARM1 antibody was used, which recognized both WT- and dnSARM1. A part of this blot is shown in Fig. 6A. **C:** Construct expression was induced on DoD9 in plated spheroids. To assess the induced expression of tRFP, images of neurites were recorded by epifluorescence and phase contrast microscopy on DoD15 (*cf.* Fig 6B). Scale bar = 200 µm. **D:** Neurites were isolated and stained with calcein-AM 18 h later. Images were recorded by epifluorescence microscopy. Neurite fragmentation was quantified by an image analysis algorithm in neurites with or without dnSARM1 induction. Data was normalized to fully fragmented WT neurites (18 h after cut). Each data point represents a biological replicate with 10 field recorded from 3-5 technical replicates. Neurite fragmentation is shown in Fig. 6F. ** p < 0.01 by Student’s t-test.


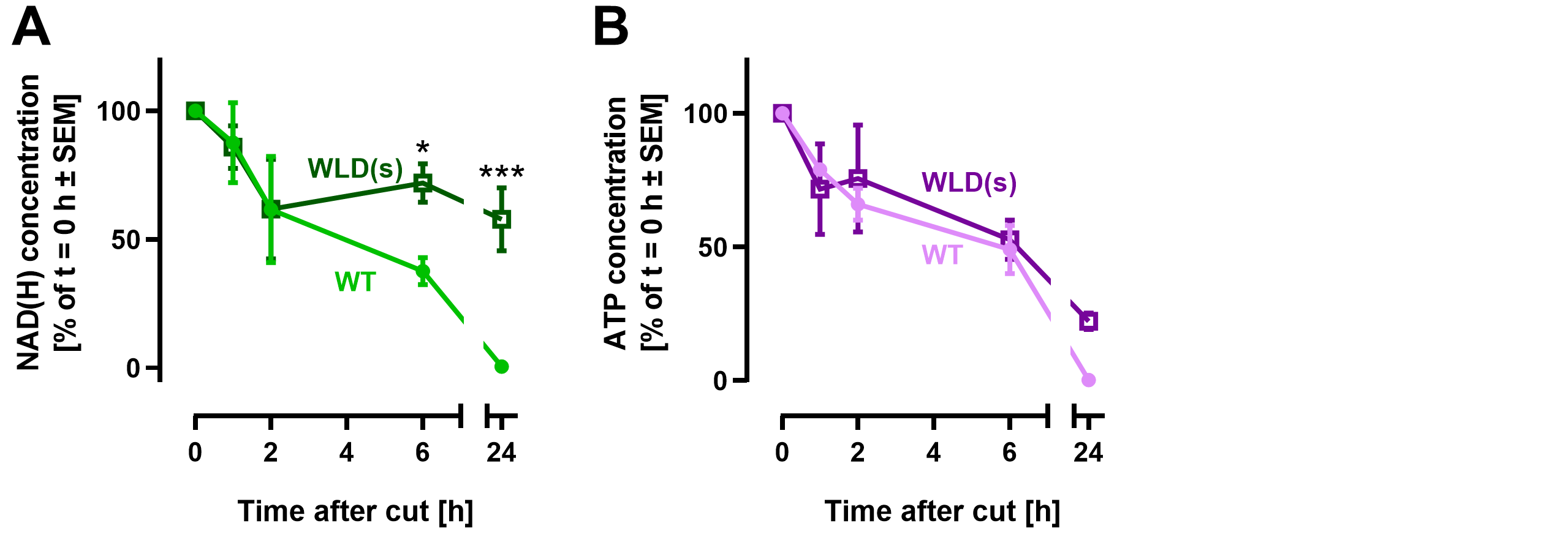


**Supplementary figure 7: ATP and NAD levels in WLD(s)-expressing neurites.** LUHMES cells were lentivirally transduced to express the “Wallerian degeneration slow” (WLD(s)) fusion protein (Schildknecht *et al.* 2013). Spheroids were generated from WT and WLD(s)-expressing cells. Spheroids were plated on DoD9. On DoD15, axotomy was induced and neurites were isolated. Neurites were lysed at different time points after axotomy and (A) the total pool of NAD^+^/NADH (NAD(H)) and (B) ATP were measured. Data was normalized to the metabolite concentrations measured in freshly isolated neurites of the respective genotype (t = 0 h). The data for 24 h after cut is also shown in Fig. 7E,F. * = p < 0.05, *** = p < 0.001 by ANOVA with Dunnet’s *post hoc* test.

**Table S1: Chemicals and media supplements used in this study.**

| **Compound** | **Supplier** | **Town** | **State** | **Country** | **Cat. no.** |
| --- | --- | --- | --- | --- | --- |
| 2-[4-(2-hydroxyethyl)piperazin-1-yl]ethanesulfonic acid (HEPES) | Sigma-Aldrich | St. Louis | MO | USA | H3375 |
| 3-[(3-Cholamidopropyl)dimethylammonio]-1-propanesulfonate (CHAPS) | Sigma-Aldrich | St. Louis | MO | USA | 220201 |
| 4-(2-Amino-ethyl)benzenesulfonyl fluoride hydrochloride (AEBSF) | Sigma-Aldrich | St. Louis | MO | USA | A8456 |
| AC-DEVD-Afc | Cayman Chemicals | Ann Arbour | MN | USA | 14459 |
| Antimycin A | Sigma-Aldrich | St. Louis | MO | USA | A8674 |
| Basic fibroblast growth factor (bFGF) | R&D Systems | Minneapolis | MN | USA | 4114-TC |
| Blasticidin | Invivogen | Toulouse |  | France | ant-bl |
| Calcein-AM | Biomol | Hamburg |  | Germany | Cay14948 |
| Carbonyl cyanide p-(trifluoro-methoxy) phenyldrazoe (FCCP) | Sigma-Aldrich | St. Louis | MO | USA | C-2920 |
| Dibutyryl-cyclic adenosine monophosphate (db-cAMP) | Sigma-Aldrich | St. Louis | MO | USA | D0627 |
| Dithiothreitol (DTT) | Carl Roth | Karlsruhe |  | Germany | 6908 |
| Ethylene glycol-bis(β-aminoethyl ether)-N,N,N′,N′-tetraacetic acid (EGTA) | Sigma-Aldrich | St. Louis | MO | USA | 324626 |
| Fetal Bovine Serum (FBS) | PAA Laboratories | Coelbe |  | Germany | A15-751 |
| Fibronectin (Fbn) | Sigma-Aldrich | St. Louis | MO | USA | F-1141 |
| FK866 | Selleckchem | Cologne |  | Germany | S2799 |
| Glia-derived neurotrophic factor (GDNF) | R&D Systems | Minneapolis | MN | USA | 212-GD |
| Glutor | Sigma-Aldrich | St. Louis | MO | USA | SML2765 |
| Glutor | Sigma-Aldrich | St. Louis | MO | USA | SML2765 |
| Hoechst-33342 | Merck | Darmstadt |  | Germany | 14533 |
| L- Glutamine | Thermo Fisher | Waltham | MA | USA | G7513 |
| Magnesium chloride | Merck | Darmstadt |  | Germany | 105833 |
| Matrigel | Corning | Corning | NY | USA | 354234 |
| N2 medium supplement | Thermo Fisher | Waltham | MA | USA | 17502048 |
| Nicotinamide | Sigma-Aldrich | St. Louis | MO | USA | N0636 |
| Nicotineamide andenine dinucleotide (NAD+) | Merck | Darmstadt | MO | USA | 481911 |
| Nicotinic acid | Calbiochem | Ann Arbor | MI | USA | 480354 |
| Oligomycin | Sigma-Aldrich | St. Louis | MO | USA | O-4876 |
| Poly-L-ornithine (PLO) | Sigma-Aldrich | St. Louis | MO | USA | P-3655 |
| Rotenone | Sigma-Aldrich | St. Louis | MO | USA | R-8875 |
| Tamoxifen | Sigma-Aldrich | St. Louis | MO | USA | T5648 |
| Tetracycline | Sigma-Aldrich | St. Louis | MO | USA | T-7660 |
| Tetramethylrhodamine (TMRE) | Sigma-Aldrich | St. Louis | MO | USA | 87917 |
| Triton-X100 | Sigma-Aldrich | St. Louis | MO | USA | T8787 |
| Trypsin-EGTA | Thermo Fisher | Waltham | MA | USA | 25300062 |
| Vacor | Sigma-Aldrich | St. Louis | MO | USA | S668923 |

**Table S2: List of oligonucleotides used for the creation of dnSARM1.** All oligonucleotides are depicted 5’ to 3’. Restriction enzymes used to clone the dnSarm1 gene (SexAI, NsiI, MfeI and NheI) are underlined. The start and the stop codon are in bold lettering.

| **Name** | **Sequence** |
| --- | --- |
| UBI-SARM-33540875 | 5’-ACCTGGTGCTCCGTCTTAGAGGTGGC**ATG**GTCCTGACGCTGCTTC-3’ |
| SARMTAA-33540874 | 5’-ATGCATCGTACGGATCC**TTA**GGTTGGACCCATGGGTG-3’ |
| MFE-ATG-33540870 | 5’-CAATTGTGGCCTCCTGGTATTCGGCGGACCACTTGATACCGTTG-3’ |
| MFE-TAA-33540871 | 5’-CAATTGAGAAGATCATCCGC-3’ |
| NHE-ATG-33540872 | 5’-GCTAGCCCTGAACATGTGCTCCAAGATG-3’ |
| NHE-TAA-33540873 | 5’-GCTAGCGAGGAGACATGCCAGAGGC-3’ |

**Table S3: List of antibodies used in this study.**

| **Target** | **Species** | **Dilution** | **Supplier** | **Cat. no.r** |
| --- | --- | --- | --- | --- |
| αII-Spectrin | mouse | 1:1000 | Merck | MAB1622 |
| α-Tubulin | rabbit | 1:5000 | Cell Signaling | 2125 |
| β-Actin | mouse | 1:5000 | Sigma-Aldrich | A5228 |
| Cytochrome C | mouse | 1:1000 | BD bioscience | 556432 |
| SARM1 | rabbit | 1:2000 | Cell Signaling | 13022 |
| NMNAT2 | mouse | 1:1000 | Santa Cruz | sc-515206 |
| βIII-Tubulin | mouse | 1:500 | BioLegend | 801202 |
| Anti-mouse-IgG-HRP | goat | 1:10 000 | Jackson | 115-036-068 |
| Anti-rabbit-IgG-HRP | goat | 1:10 000 | Biozol | VEC-PI-1000 |
| Anti-mouse-IgG1-Alexa-488 | goat | 1:1000 | Invitrogen | A21121 |
| Anti-mouse-IgG2a-Alexa-647 | goat | 1:1000 | Invitrogen | A21241 |
