# Supplementary figures and images for: Programmed neurite degeneration in human central nervous system neurons driven by changes in NAD+ metabolism
Source: Cell Death Dis. 2025 Jan 17;16(1):24. doi: 10.1038/s41419-024-07326-w (PMC11742042; doi:10.1038/s41419-024-07326-w)

Fig.1D:

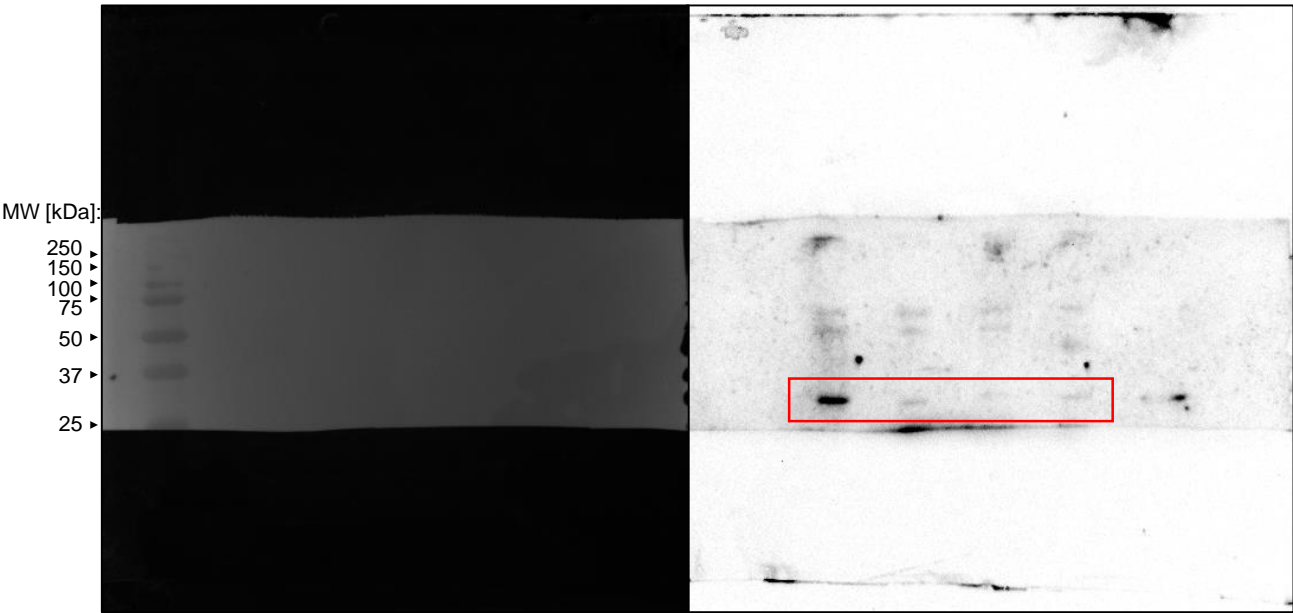

NMNAT2

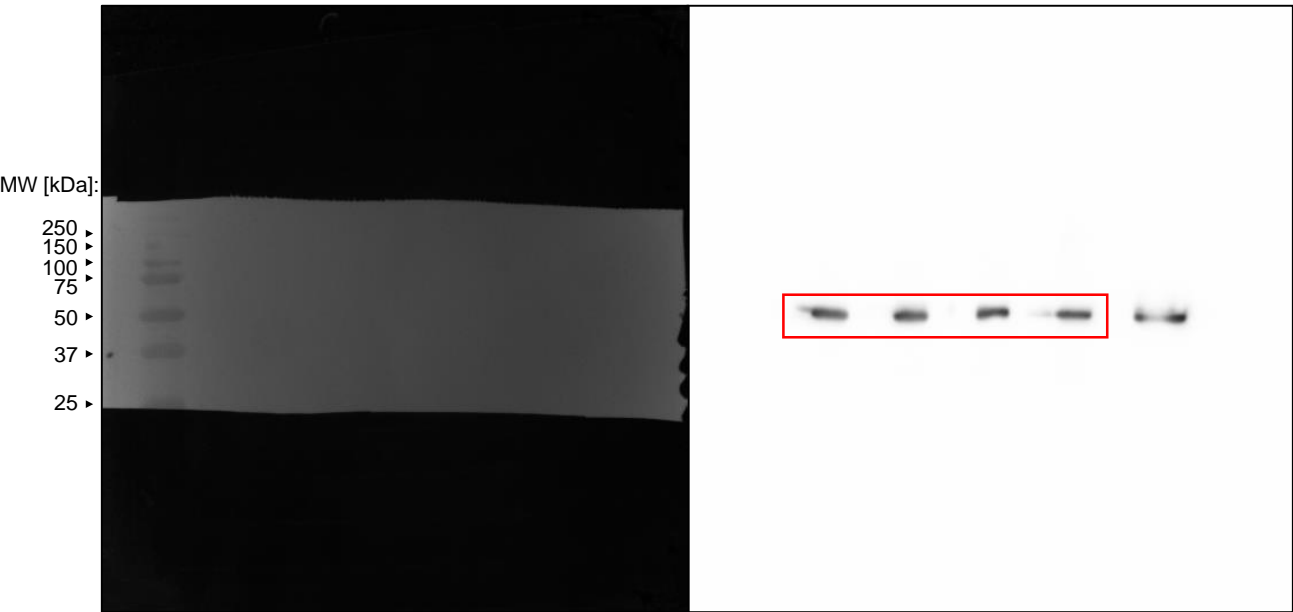

$\alpha$ -Tubulin

Fig.3D:

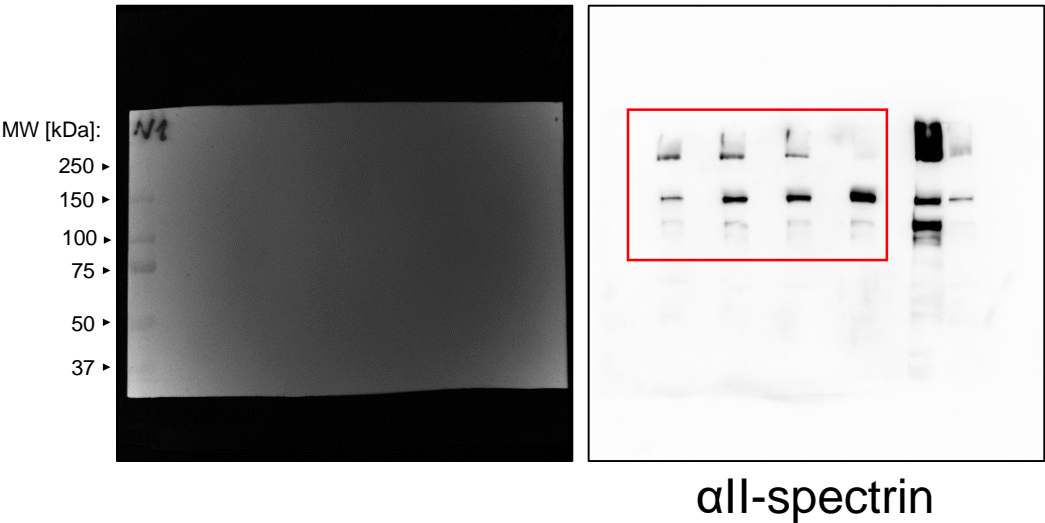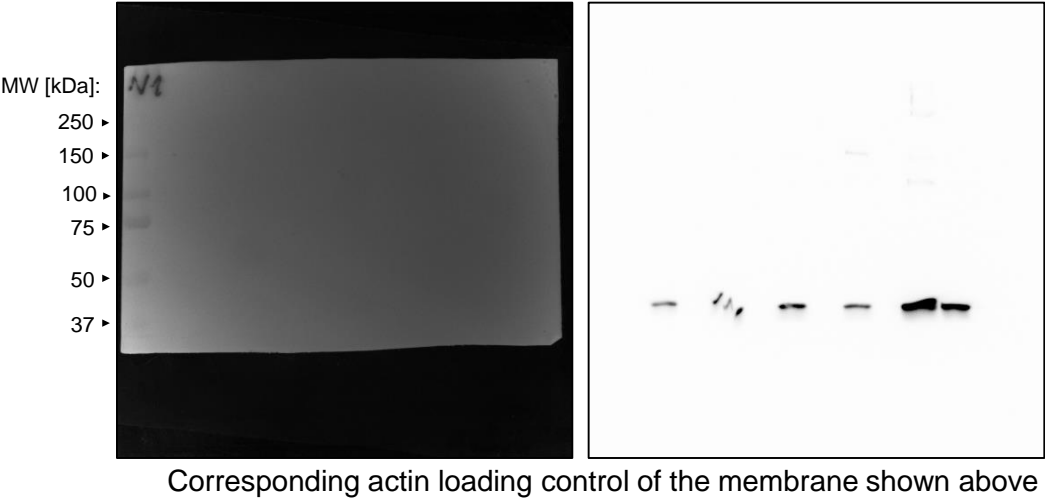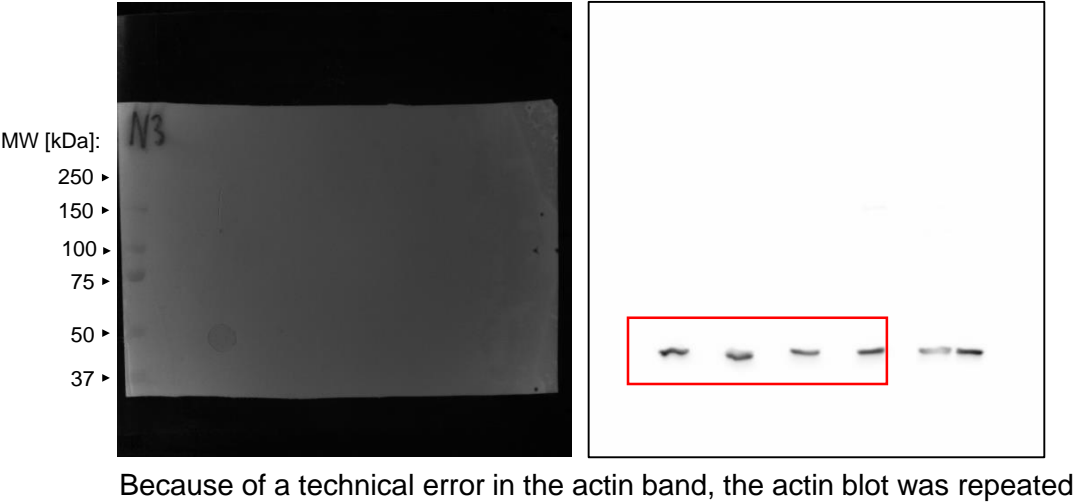

Fig. 3E:

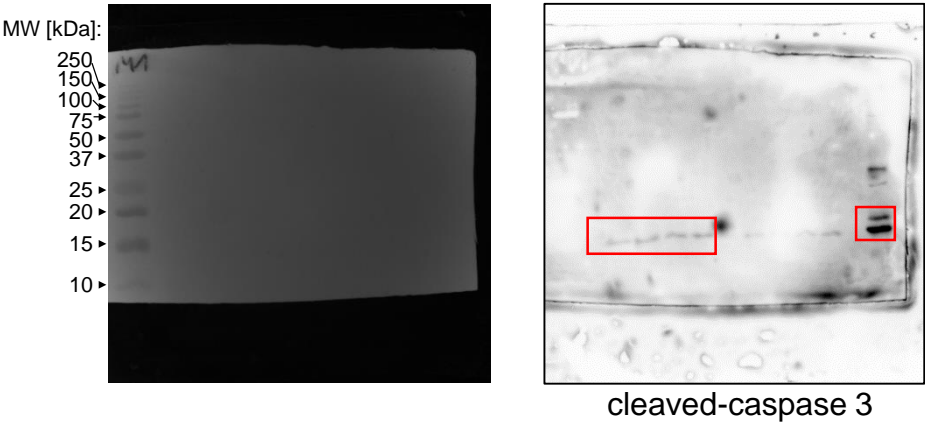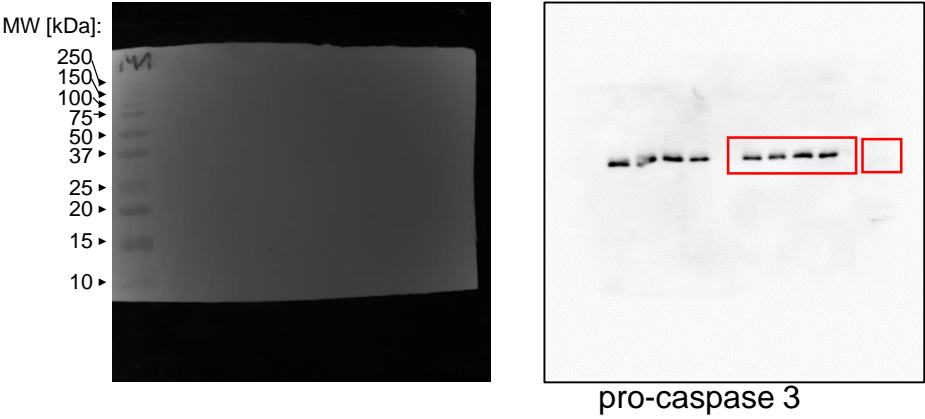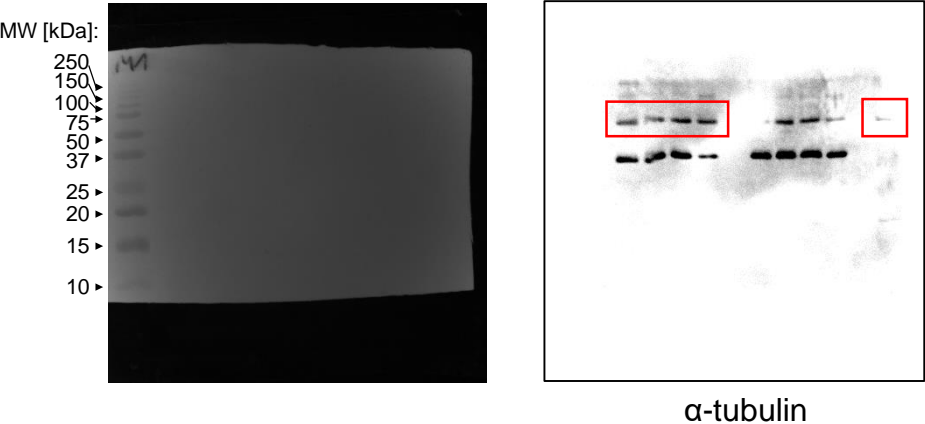

Fig. 6A, Fig S6B

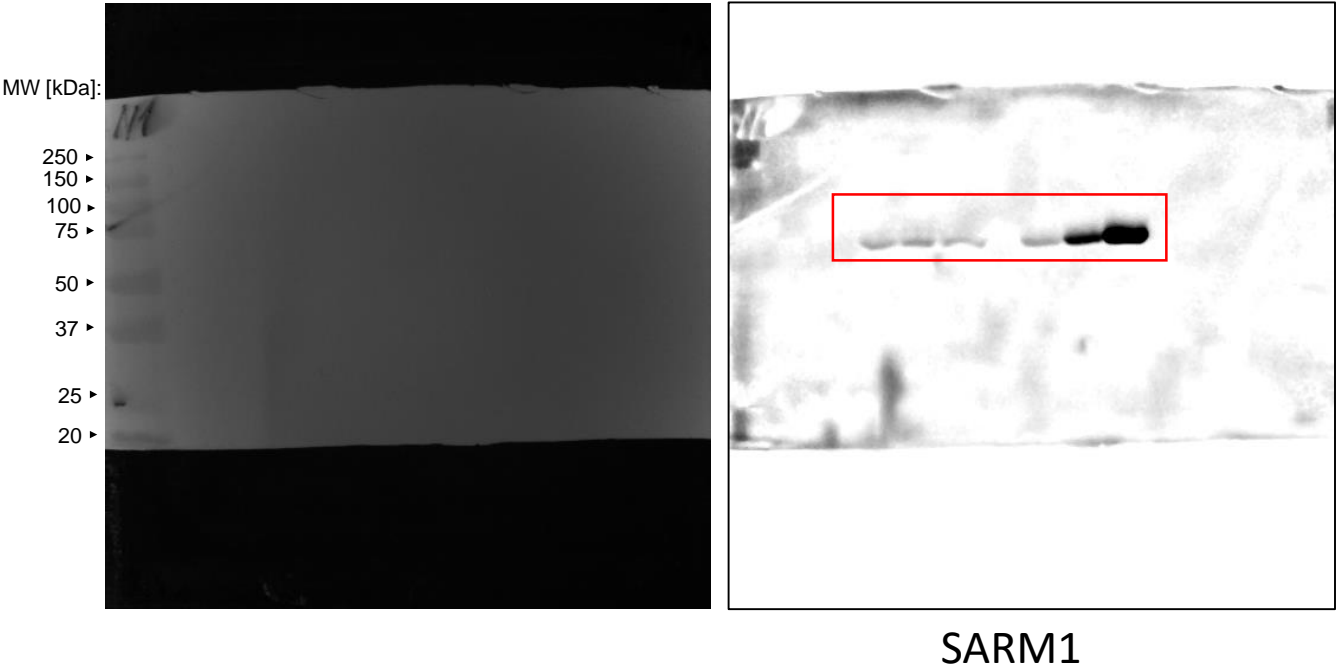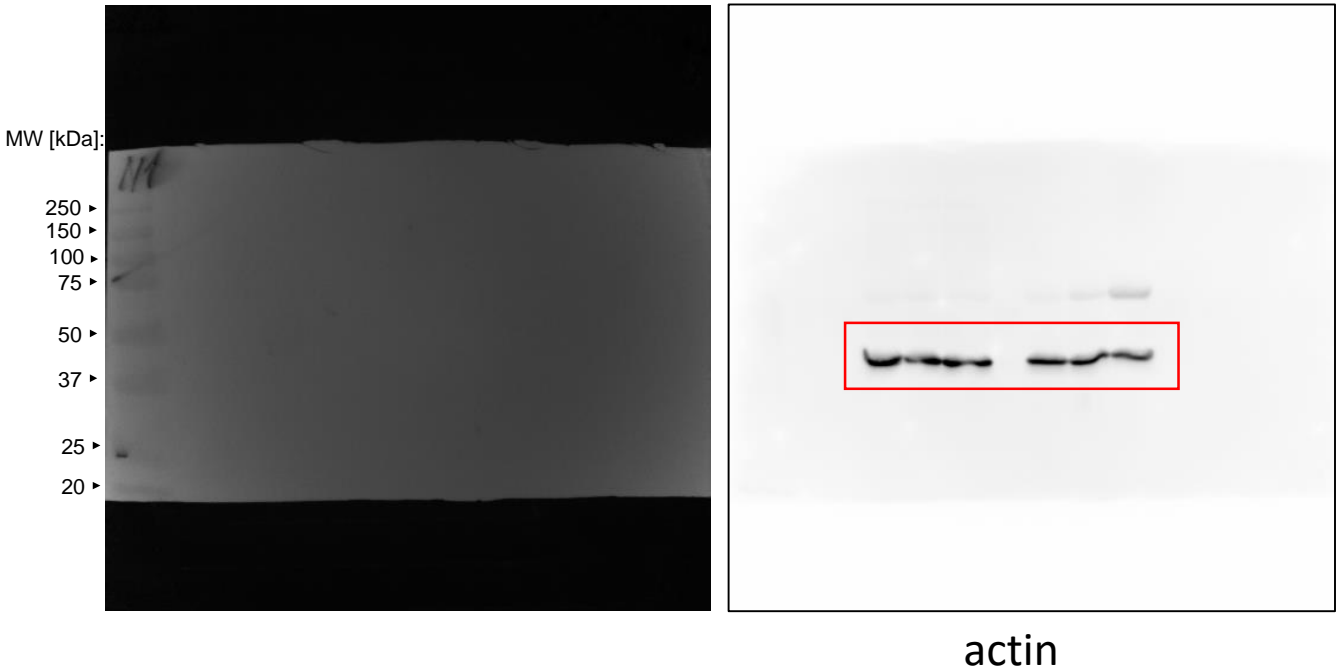

Fig. S4C

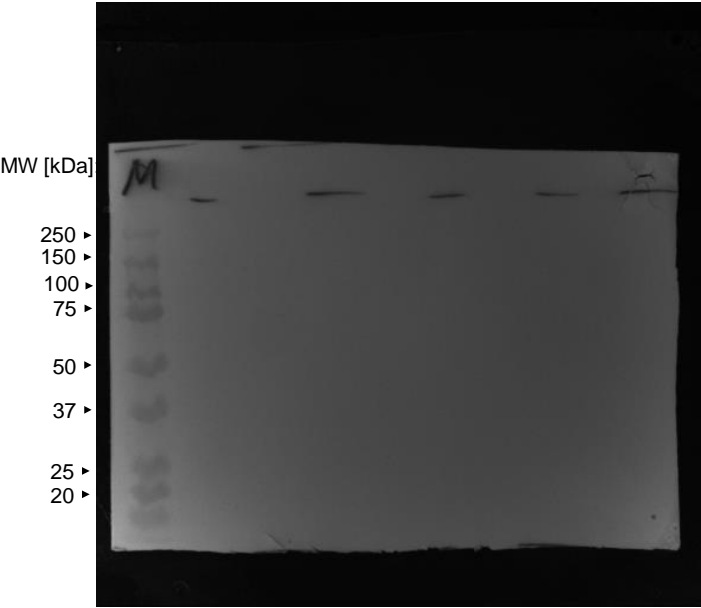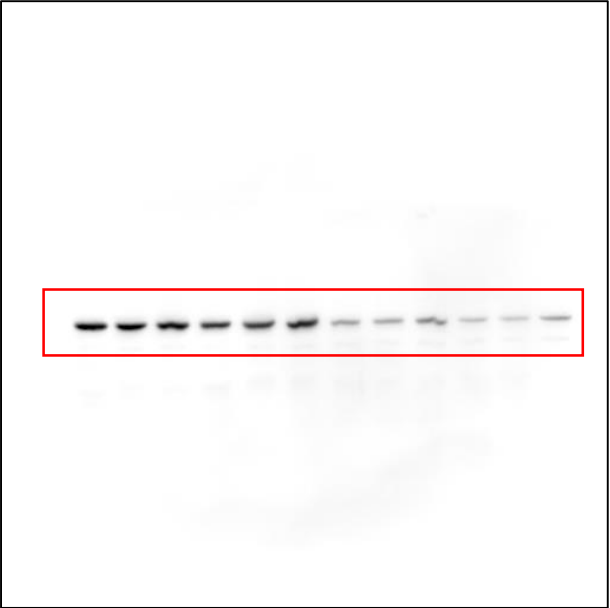

SARM1

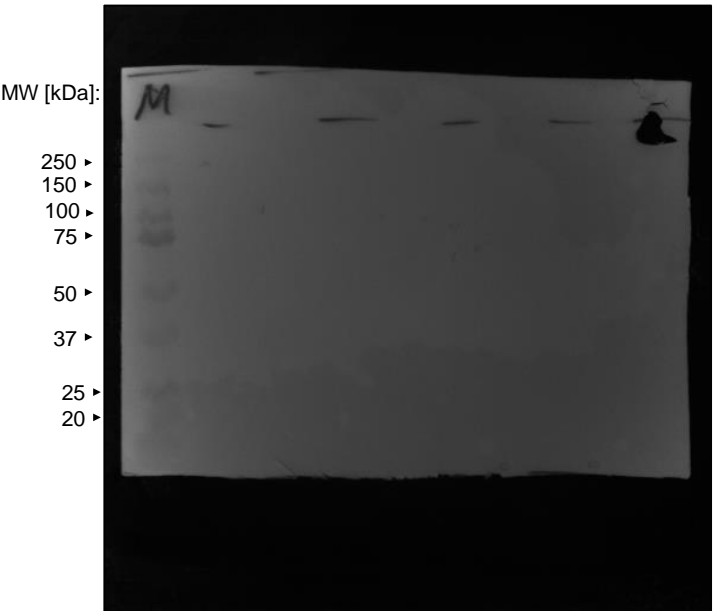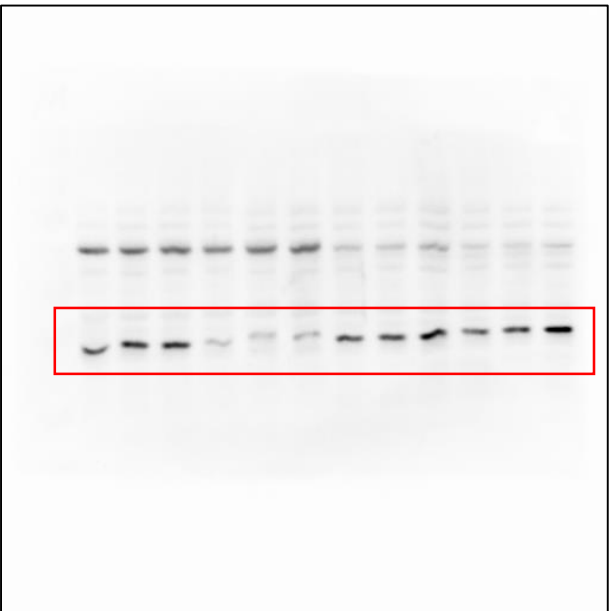

GADPH

Fig. S5B

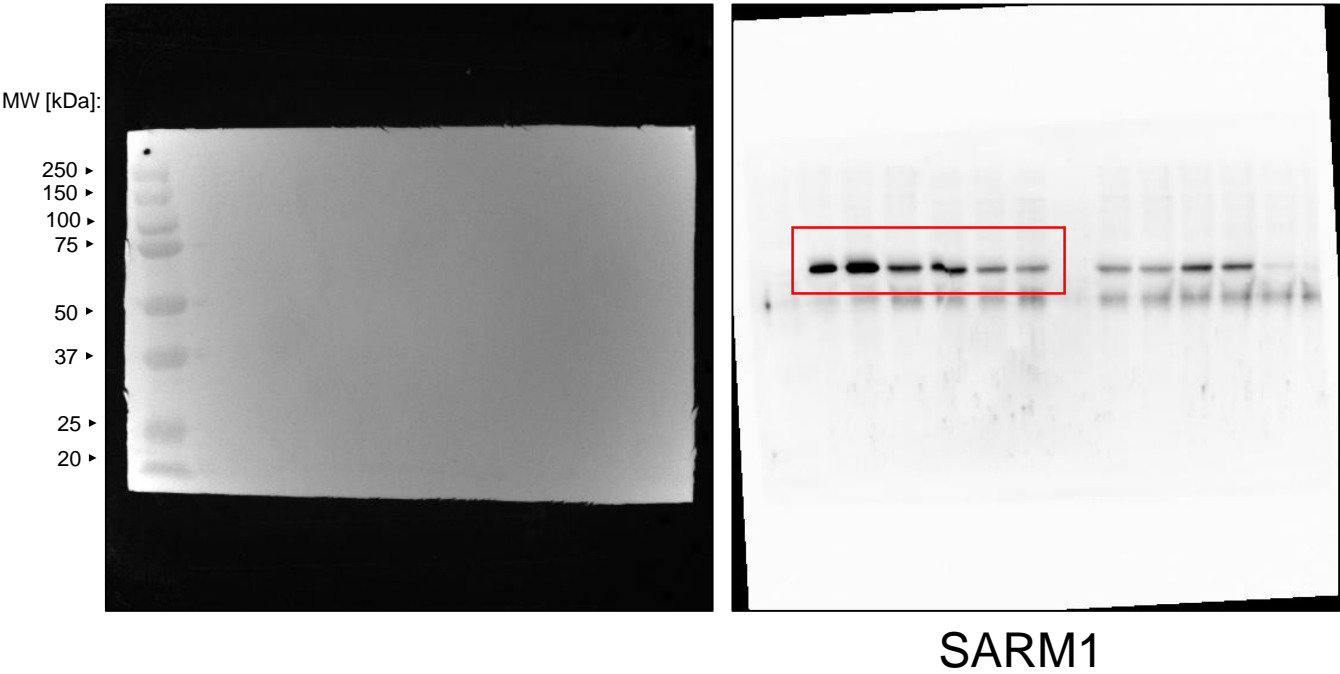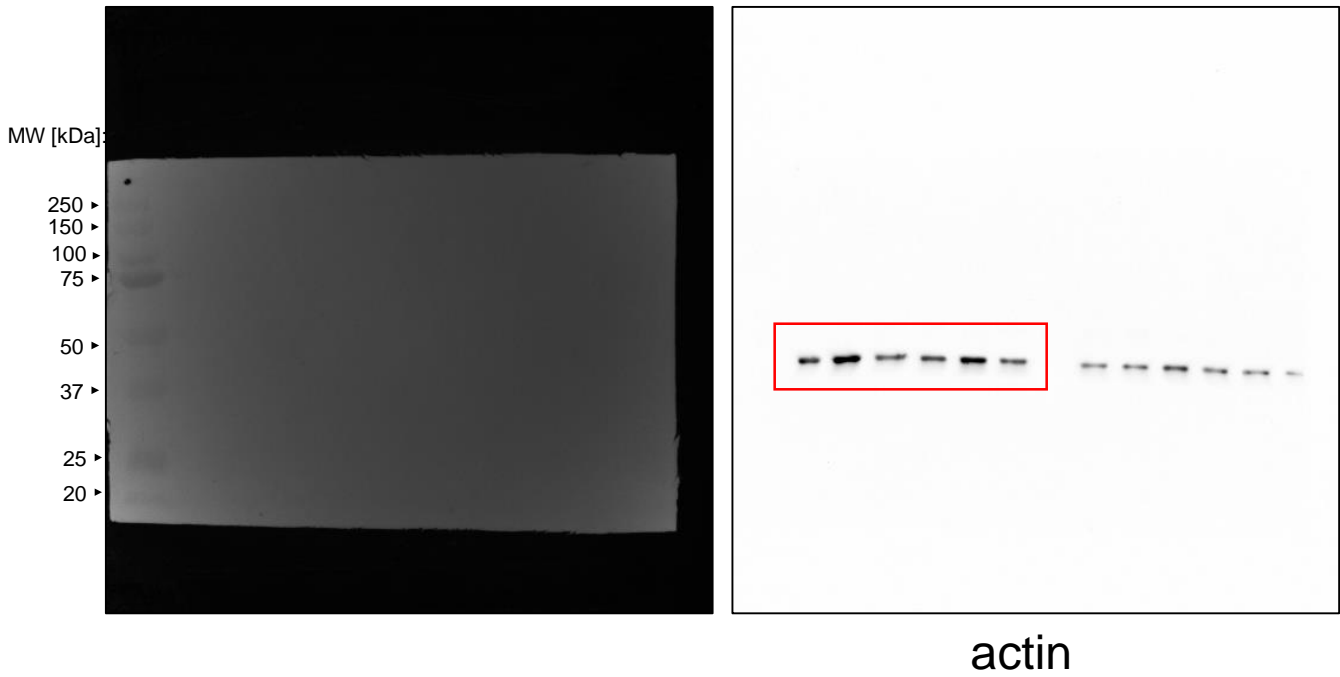

Supplement: Supplementary file 2 — Data Set 1 [file 41419_2024_7326_MOESM2_ESM.pdf]
